# Preparation and Performance Evaluation of a Lost-Circulation-Control Gel for Fractured Formations

**DOI:** 10.3390/gels12080702

**Published:** 2026-08-05

**Authors:** Yundong Zheng, Xiaojiang Qiu, Zhaocai Yu, Fan Xiao, Tianan Deng, Peng Xu, Lei Pu, Jingwei Liu

**Affiliations:** 1 PetroChina Southwest Oil & Gas Field Company, Chengdu 610051, China; zhengyd1987@petrochina.com.cn (Y.Z.); qiuxiaojiang@petrochina.com.cn (X.Q.); yuzhaocai@petrochina.com.cn (Z.Y.); xiaofan01@petrochina.com.cn (F.X.); dengtiana223@petrochina.com.cn (T.D.); 2 School of Petroleum Engineering, Yangtze University, Wuhan 430100, China; puleigogo@163.com

**Keywords:** fractured formation, drilling lost circulation, wellbore instability, lost-circulation-control gel, acrylamide copolymer

## Abstract

To address the dual challenges of severe lost circulation and wellbore instability in fractured formations, an acrylamide-based lost-circulation-control gel (DF-PG) was synthesized via aqueous-solution free radical polymerization using acrylamide (AM) and sodium acrylate (SA) as monomers, N,N′-methylenebisacrylamide (MBA) as a crosslinker, and ammonium persulfate (APS) as an initiator. The optimal formulation was determined as 6.8% AM, 1.7% SA (mass ratio 4:1), 0.09% MBA, and 0.18% APS, reacted at 60 °C for 4 h. The gel achieves a 276% equilibrium swelling ratio in 20,000 mg/L simulated-formation water, with a compressive strength of 1.2 MPa and temperature resistance up to 120 °C. For fractured cores with 0.5–2.0 mm fracture widths, its sealing efficiency exceeds 95% with breakthrough pressure above 1.2 MPa, and the sealing-efficiency-retention rate remains over 85% after 72 h of scouring. DF-PG realizes the integrated functions of lost circulation control and wellbore stabilization through an “infiltration-swelling-filling“ mechanism, providing a novel technical solution for drilling operations in fractured formations.

## 1. Introduction

Fractured formations are typical complex and hard-to-drill strata in petroleum drilling engineering, which are widely distributed in fold belts, fault fracture zones, weathered crusts, and areas with well-developed rock joints [1,2]. Such formations feature poor rock integrity, interlaced network fractures, low rock consolidation strength, and high pore connectivity [3,4]. Downhole complications including drilling fluid lost circulation, wellbore collapse, and pipe sticking commonly occur during drilling, which seriously restrict the efficiency and safety of drilling operations [5,6]. Fractured formations present an uneven pore pressure distribution and multi-scale fracture systems ranging from micron-scale microfractures to centimeter-scale macrofractures, accompanied by extremely low formation bearing capacity. In addition, loose rock masses are prone to secondary collapse under the scouring of drilling fluid and in situ stress, which further expands the scope of lost-circulation channels [7].

Conventional bridging lost-circulation materials only form superficial bridging at the openings of macrofractures and fail to penetrate into microfracture networks effectively [8,9]. Meanwhile, the formed plugging layer exhibits weak adhesion to fractured rock masses and is easily washed away by flowing drilling fluid [10]. Although cement slurry possesses high mechanical strength for lost-circulation control, its thickening time is difficult to regulate. Problems such as slurry leakage and weak interfacial bonding between consolidated bodies and host formations frequently occur, making it impossible to achieve full-range tight plugging of fractured formations [11].

Lost circulation and wellbore instability are two core challenges for drilling in fractured formations, and the two issues interact to form a vicious cycle. Drilling fluid loss leads to a sharp drop in hydrostatic pressure in the wellbore, which cannot balance the formation of in situ stress and eventually triggers wellbore collapse. In turn, loose cuttings generated by wellbore collapse block the circulation passages of drilling fluid, expand fracture leakage channels, and aggravate lost circulation [12,13].

Field statistical data from deep carbonate fractured reservoirs in the Sichuan-Chongqing area, clastic fractured reservoirs in Northwest China, and weathered crust formations in East China demonstrate that the comprehensive occurrence rate of lost circulation and wellbore instability during drilling in fractured formations reaches 62–75% [14,15]. However, the comprehensive success rate of conventional technologies such as bridging lost-circulation control and cement slurry sealing is only 38–47% [16]. This has become a critical bottleneck restricting the exploration and development of oil and gas in complex formations [17,18].

Currently, three main types of technologies are applied for lost-circulation control and wellbore stabilization in fractured formations. The first category is a conventional bridging lost-circulation-control technology. Different granular bridging materials including walnut shells, fibers, and mica are compounded to form plugging layers at thief zones. Nevertheless, this technology shows a limited plugging effect on microfracture networks, and the low-strength plugging layers cannot resist the combined action of in situ stress and drilling fluid erosion. The second category is chemical consolidation lost-circulation-control technology. Chemical grouts such as polyurethane and epoxy resin are adopted for grouting and consolidation, which can fill microfractures and consolidate loose rock masses. However, these grouts suffer from short curing times, poor injectability, and high costs and thus are not applicable to large-scale fractured formations. The third category is the composite technology combining wellbore strengthening and lost-circulation control. Drilling fluid modification is adopted to improve wellbore inhibition performance, and lost-circulation materials are used for auxiliary plugging. This technology can only alleviate wellbore instability rather than fundamentally solve the lost-circulation problem in fractured formations [19,20,21].

**Figure 1:**
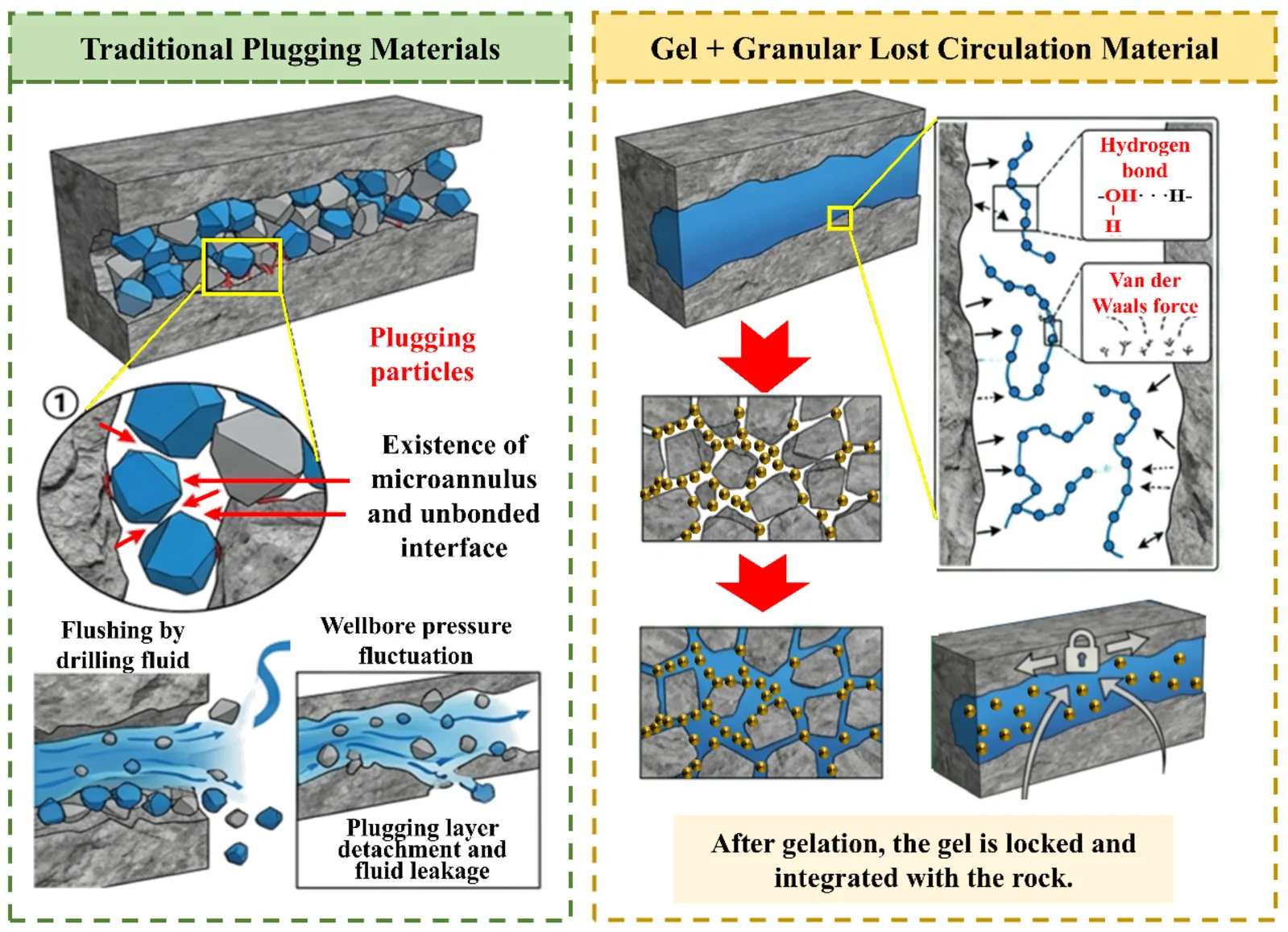
Working mechanism of gel-based lost-circulation-control materials.

With favorable rheological properties, adaptive filling performance, elastic deformability, and rock adhesion, gel-based lost-circulation materials have become a key research direction to address the dual problems of lost circulation and wellbore instability in fractured formations [22,23]. In the liquid state, these materials possess excellent injectability and can flow into the microfracture networks and rock voids of fractured formations. After gelation, the formed three-dimensional network structure presents outstanding elasticity and adhesion. It can not only fill leakage channels for plugging, but also consolidate loose cuttings to improve the overall strength of rock masses, realizing the integration of lost-circulation control and wellbore stabilization [24]. As shown in Figure 1.

According to different polymerization monomers and crosslinking modes, drilling lost-circulation gels are divided into acrylamide-based, polyvinyl alcohol (PVA)-based, xanthan gum-based, and other systems. Among them, acrylamide-based gels stand out as a research hotspot for lost-circulation control and wellbore stabilization in fractured formations, due to their flexible polymerization process, controllable crosslinked network structure, good rock adhesion, and low cost [25,26].

In the field of lost-circulation control for oil and gas wells, conventional lost-circulation materials (LCMs) such as foams, rigid granular agents, and ordinary polymer materials suffer from distinct drawbacks. To address these deficiencies, numerous researchers have developed various modified gel systems for targeted applications. As shown in Table 1. Jia [27] fabricated nanosilica-reinforced composite gels, which feature rapid gelation, high pressure-bearing capacity, facile degradability, and low formation damage and are suitable for ultra-low-pressure fractured reservoirs, yet their temperature tolerance remains limited. Guo [28] constructed a thixotropic gel crosslinked via multiple non-covalent bonds; the reversible network endows the gel with shear self-recovery performance and strong rock adhesion, rendering it applicable for lost-circulation mitigation in high-temperature sand formations. Chen [13] developed a hydrophobically modified gel with a dual crosslinking system, which significantly improves long-term thermal stability at 140 °C and enables the effective plugging of wide fractures, serving as a viable alternative to cement for temporary plugging operations. Du [29] proposed a thermoresponsive supramolecular gel free of crosslinkers and gel breakers, which undergoes reversible sol–gel–sol phase transitions triggered by temperature, facilitating convenient construction and imposing minimal formation damage.

Nevertheless, most existing plugging gels are prefabricated, and their application in fractured formations is subject to prominent limitations [30]:

(1) Delivered as high-viscosity bulk gels, they fail to penetrate micro-fracture networks;
(2) Their flow behavior is highly susceptible to downhole environmental variations, which may trigger premature gelation and consequently lead to plugging failure;
(3) The load-bearing strength of such gels is insufficient to withstand the in situ stress of fractured formations, so the formed plugging zone is prone to failure under forward or back differential pressure.

In view of the above limitations, a novel lost-circulation gel DF-PG was prepared via aqueous-solution free radical polymerization using acrylamide and sodium acrylate as comonomers, targeting the characteristics of lost circulation and wellbore instability in fractured formations. The monomer ratio and crosslinked structure were optimized to endow the gel with high elasticity, strong adhesion, and favorable temperature resistance. A multi-dimensional performance test system specially for fractured formations was established, covering the swelling property, temperature resistance, mechanical strength, and plugging performance, with detailed test procedures formulated. The synergistic effect between the gel and rigid lost circulation particles was systematically studied, and effective plugging for multi-scale fractures with widths of 0.5–5.0 mm was achieved. Meanwhile, the four-stage working mechanism of the infiltration–swelling–filling–consolidation of the gel was revealed. This work provides a new technical scheme and experimental support for the comprehensive management of lost circulation and wellbore stability during drilling in fractured formations.

## 2. Results and Discussion

### 2.1. Chemical Structure and Apparent Performance Characterization of the Gel

#### 2.1.1. Fourier Transform Infrared Spectroscopy Analysis

**Figure 2:**
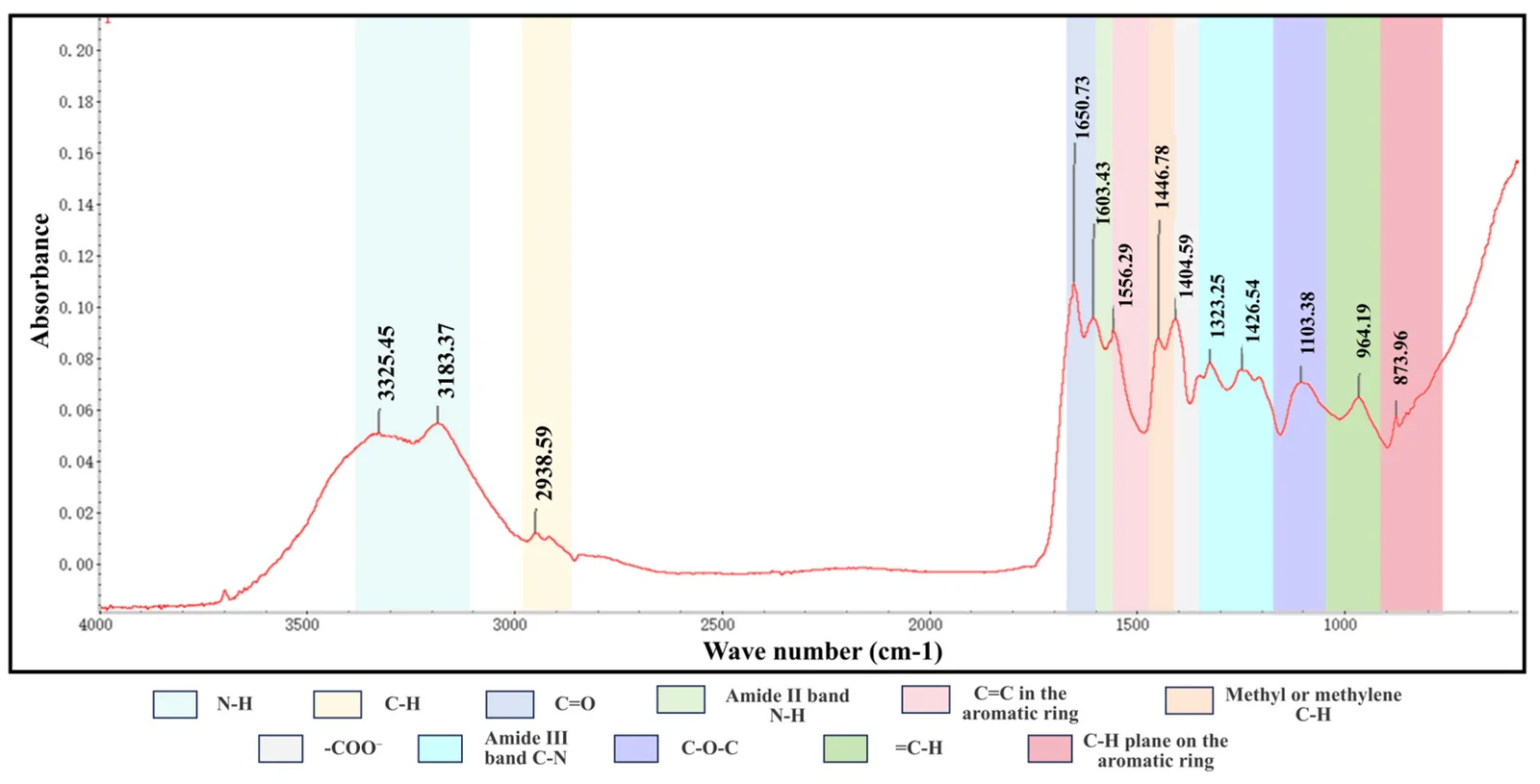
FTIR spectroscopy analysis of gel DF-PG.

The FTIR spectrum of the DF-PG xerogel is shown in Figure 2. Key characteristic absorption peaks confirm the successful copolymerization of AM and SA and the formation of a three-dimensional crosslinked network.

The broad absorption peaks at 3325 cm^−1^ and 3183 cm^−1^ correspond to the N–H stretching vibration of amide groups from AM units, and the broad peak shape indicates extensive hydrogen bonding among molecular chains, which provides the structural basis for the water absorption and swelling performance of the gel. The absorption peak at 2948 cm^−1^ is attributed to the antisymmetric stretching vibration of –CH_2_– groups on the polymer backbone, reflecting the formation of a long-chain aliphatic polymer structure.

The strong absorption peak at 1650 cm^−1^ is the characteristic C=O stretching vibration of the amide I band, and the peak at 1556 cm^−1^ corresponds to the N–H bending vibration coupled with C–N stretching of the amide II band, both confirming the existence of acrylamide units in the copolymer. The absorption peak at 1404 cm^−1^ is assigned to the symmetric stretching vibration of carboxylate groups (–COO^−^) from sodium acrylate units, verifying the successful introduction of SA into the copolymer chain. These characteristic peak assignments are consistent with those reported in previous studies on acrylamide–sodium acrylate copolymer hydrogels. For instance, Xu et al. observed similar amide I (1652 cm^−1^) and amide II (1554 cm^−1^) bands in an AM/AA-based plugging gel, and the carboxylate symmetric stretching peak at 1406 cm^−1^ was also reported by Jia et al. in nanocomposite hydrogels. The slight wavenumber shifts (within 2–5 cm^−1^) observed in DF-PG compared with literature values can be attributed to differences in the monomer feed ratio and crosslinking density. The hydrogen-bond broadened peaks at 3325 cm^−1^ and 3183 cm^−1^ also match the spectral features of polyacrylamide crosslinked networks summarized in the review of Fang et al. Overall, the FTIR spectral features of DF-PG agree well with established data for poly (acrylamide-co-sodium acrylate) networks, corroborating the successful synthesis of the target copolymer gel.

#### 2.1.2. Thermogravimetric Analysis

**Figure 3:**
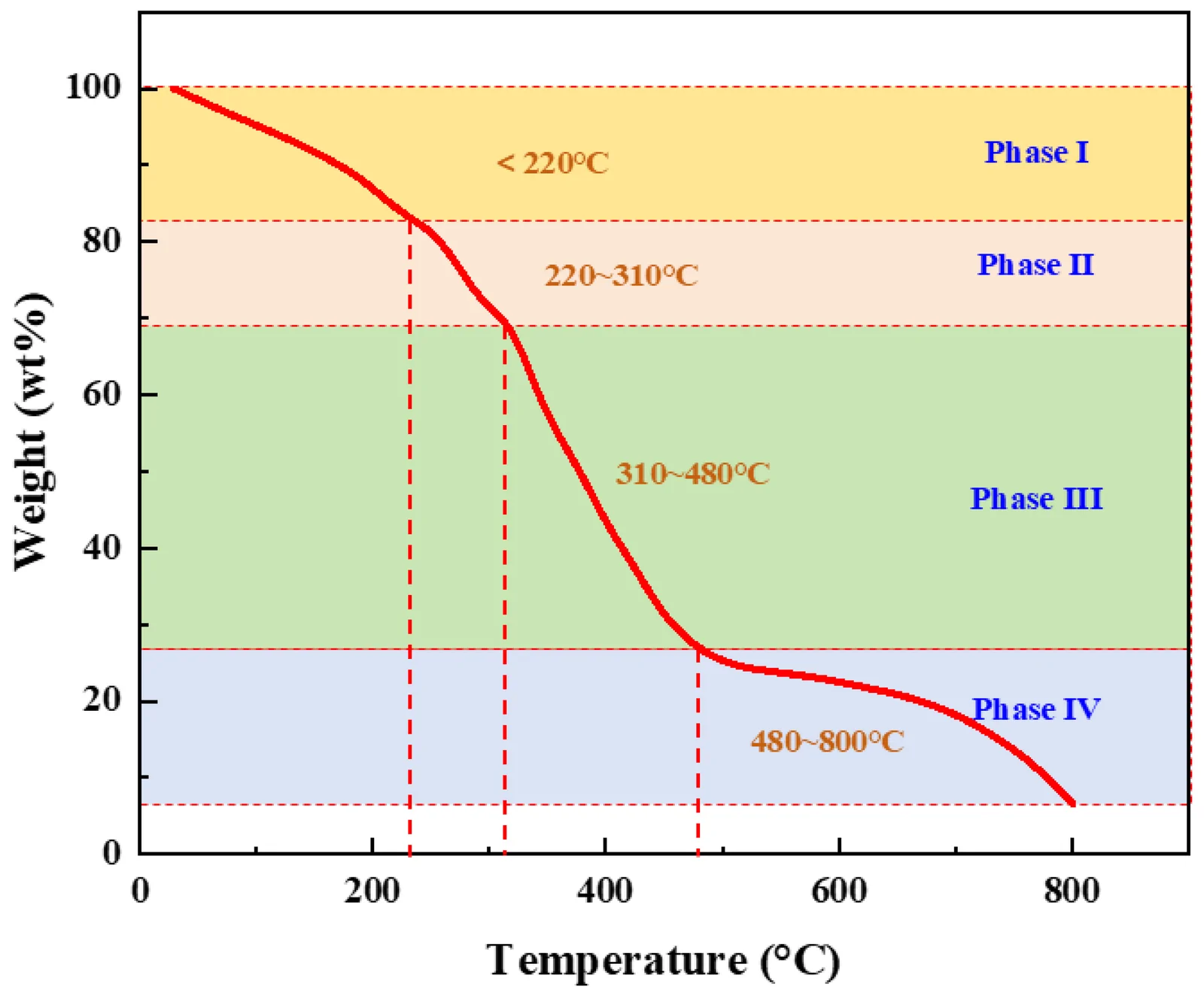
Thermogravimetric analysis curve of gel DF-PG.

The thermogravimetric analysis results indicate that the DF-PG gel exhibits 5% weight loss at approximately 202 °C. Significant decomposition of side-chain functional groups occurs mainly within the temperature range of 220–310 °C, whereas extensive degradation of the polymer backbone does not occur until temperatures exceed 310 °C. These findings demonstrate that the carbon–carbon backbone of the AM/SA copolymer maintains excellent thermal stability at 150 °C and does not undergo significant chain scission. Therefore, degradation of the polymer backbone is unlikely to be the primary cause of gel failure under these conditions. As shown in Figure 3.

Accordingly, the deterioration of the DF-PG gel at 150 °C is primarily attributed to the degradation of the crosslinked network structure rather than the thermal decomposition of the polymer backbone. Under prolonged hydrothermal conditions, the amide groups (–CONH_2_) derived from acrylamide units may undergo partial hydrolysis, leading to the formation of additional carboxylate groups and small amounts of ammonia. This hydrolysis process alters the intermolecular hydrogen-bonding interactions and enhances electrostatic repulsion between polymer chains, thereby weakening the stability of the gel network.

Simultaneously, the crosslinking junctions formed by N,N′-methylenebisacrylamide (MBA) may undergo partial hydrothermal cleavage at elevated temperatures, resulting in a gradual reduction in the effective crosslinking density. As the crosslink density decreases, the three-dimensional polymer network becomes progressively loosened, accompanied by pore enlargement and a reduction in the integrity of the network skeleton. Consequently, the mechanical strength, elastic recovery capability, and plugging stability of the gel are significantly deteriorated.

Therefore, the thermal failure of the DF-PG gel at 150 °C is mainly associated with the hydrolysis of amide groups and partial degradation of MBA-derived crosslinking sites, which collectively lead to the deterioration of the three-dimensional crosslinked network, rather than degradation of the polymer carbon–carbon backbone. This interpretation is consistent with the thermogravimetric analysis results, which show that the onset temperature of backbone decomposition is substantially higher than the investigated service temperature. These thermal-degradation characteristics are in good agreement with the literature on acrylamide-based covalently crosslinked hydrogels. Chen et al. [13] reported a 5% weight loss temperature around 198–205 °C for hydrophobically modified AM dual-crosslinking gels, and Guo et al. [28] observed that the main carbon–carbon backbone decomposition of thixotropic polyacrylamide gels only occurs above 310 °C, both of which are highly consistent with the thermal test data of DF-PG in this work. The two-stage degradation pattern—side-chain functional group decomposition (220–310 °C) followed by polymer backbone scission (>310 °C)—is a well-documented universal feature of MBA-crosslinked polyacrylamide networks proposed by Bai et al. [26]. The slight difference in the mass loss rate below 220 °C is caused by the different water-retention capacity of gel networks with distinct monomer ratios.

#### 2.1.3. Micromorphology (SEM) Analysis

Field emission scanning electron microscopy (FE-SEM) was used to observe the three-dimensional network structure, pore size distribution, and micromorphology of the gel. The SEM images were quantitatively analyzed using ImageJ software (version 1.53, National Institutes of Health, USA). For each sample, five different fields of view were selected to determine the pore size distribution, and the final results were presented as average values.

**Figure 4:**
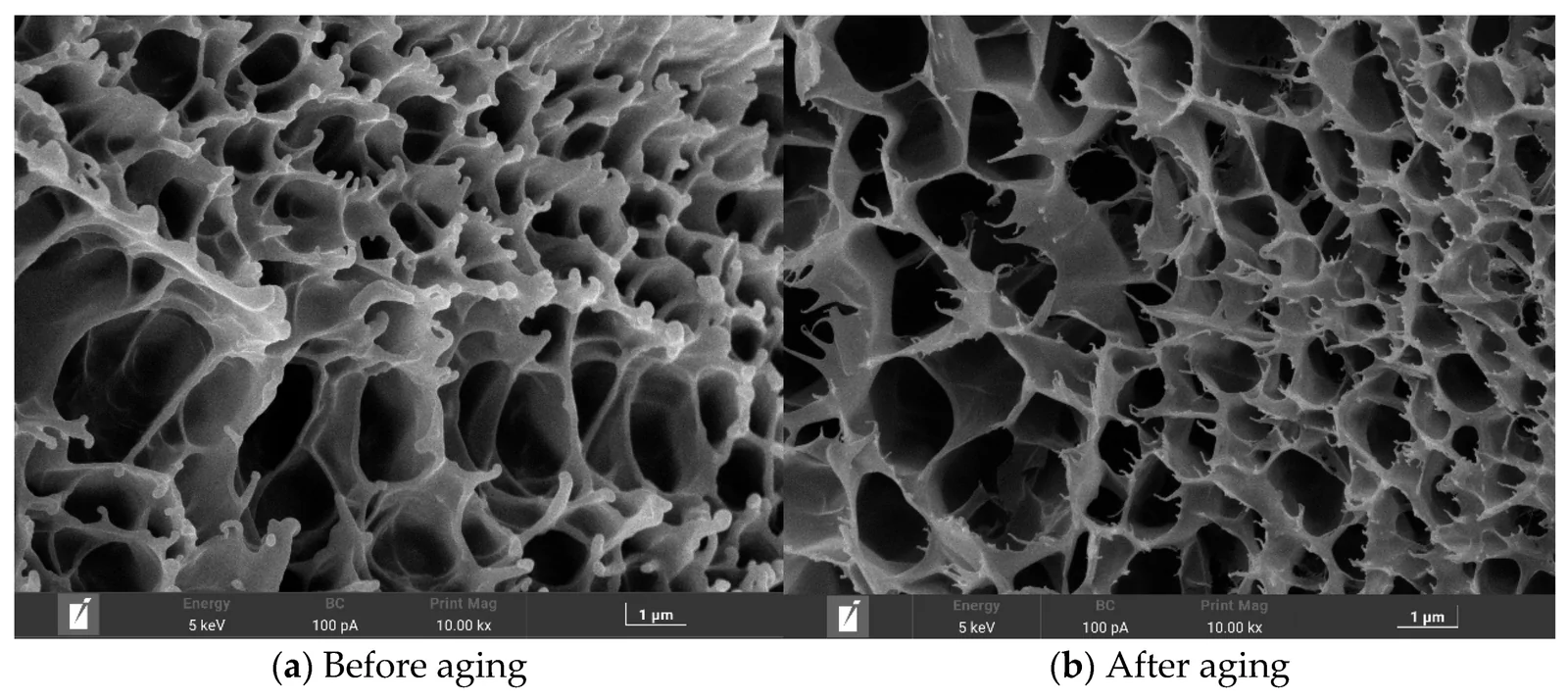
Comparison of SEM test results of the gel before and after hot rolling.

The fresh dried gel exhibits a continuous, dense, and highly interconnected three-dimensional porous network structure. The pore walls are crosslinked in flexible filamentous and lamellar forms with uniformly distributed pores. Quantitative statistics show that its average pore size is (0.82 ± 0.21) μm. The intact network skeleton lays a structural foundation for the swelling and mechanical properties of the gel. After high-temperature aging, the overall continuity and integrity of the gel three-dimensional network are well maintained, without skeleton collapse or fracture. Only local pore walls become slightly thinner, and the average pore size increases to (1.15 ± 0.34) μm. As shown in Figure 4.

Microstructural analysis reveals that the covalently crosslinked network of the DF-PG gel possesses excellent thermal stability. Only slight relaxation of molecular chains occurs at high temperature, and no cleavage of crosslinking bonds is observed. This finding is consistent with the results of the thermogravimetric analysis and macroscopic performance tests. The slight increase in pore size leads to a minor decrease in the gel swelling ratio, while the gel still retains favorable elasticity and plugging capacity. It can effectively resist the coupled thermal-shear effect in downhole environments and maintain long-term plugging performance.

### 2.2. Effect of Reaction Components on Gel Properties

#### 2.2.1. Effect of Monomer Ratio on Gel Properties

**Figure 5:**
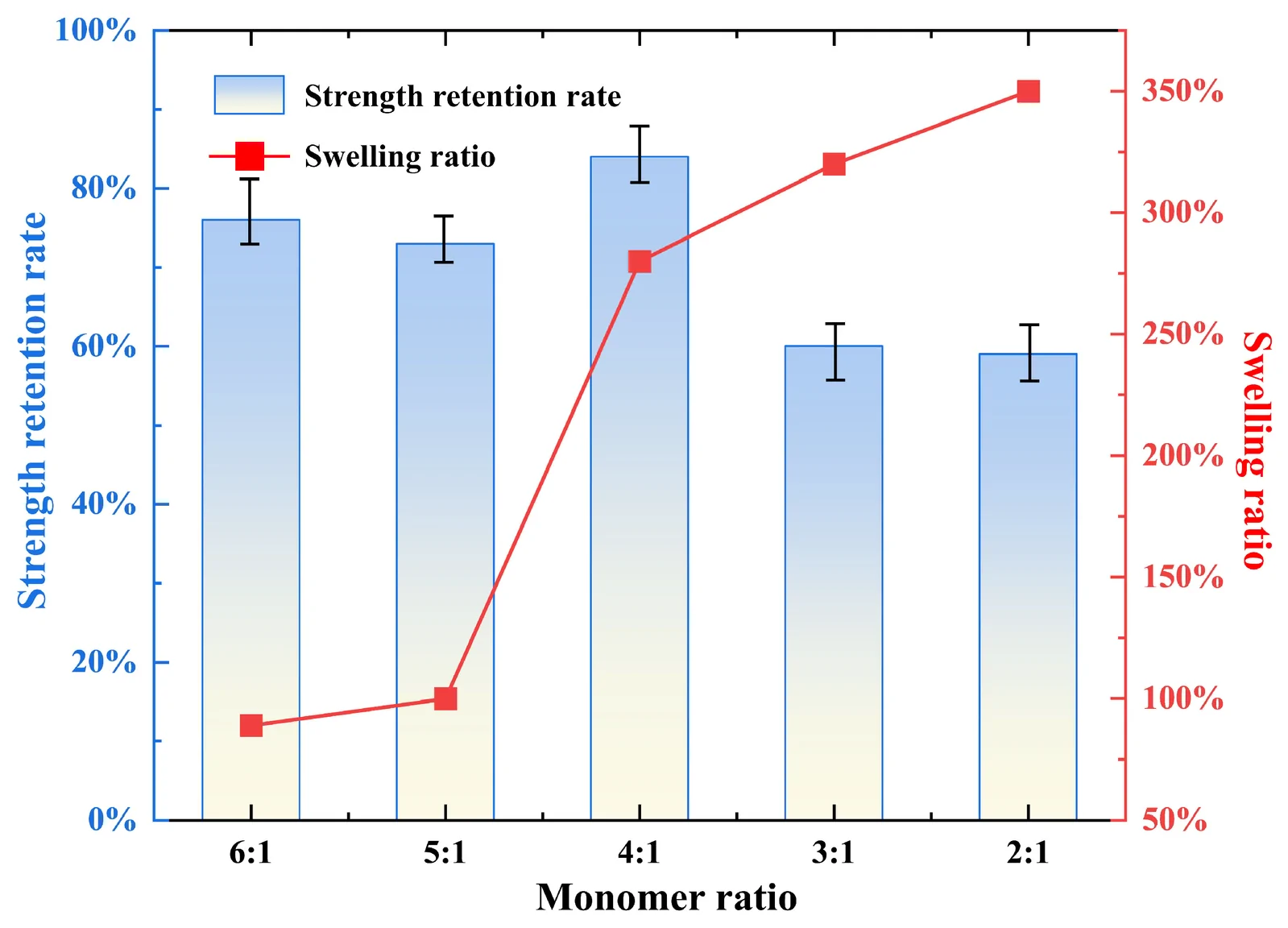
Effect of monomer ratio on gel properties.

The anti-leakage gel DF-PG for fractured formations was synthesized via aqueous-solution free radical polymerization. A single-factor variable method was adopted to optimize the formulation and preparation conditions. The comprehensive performance index (CPI) was selected as the response variable. The CPI was calculated via the weighted summation of four core indicators: compressive strength (weighting factor = 0.3), 24 h equilibrium swelling ratio (weighting factor = 0.25), performance retention rate after aging at 120 °C (weighting factor = 0.25), and plugging efficiency for fractures of a 1.0 mm width (weighting factor = 0.2). The CPI ranges from 0 to 1. The formulation corresponding to the maximum CPI value was determined as the optimal one. As shown in Figure 5.

With other reaction conditions fixed, the mass ratio of acrylamide (AM) to sodium acrylate (SA) was adjusted. The results show that the gel exhibits the optimal comprehensive performance with a balanced mechanical strength, swelling capacity, and bonding performance at an AM-to-SA mass ratio of 4:1. As the main monomer, AM contains amide groups that can form a dense hydrogen bond network and crosslinked structure, endowing the gel with excellent mechanical strength and structural stability. As a functional monomer, SA carries carboxylate groups that can significantly improve the hydrophilicity and swelling-filling capacity of the gel. Meanwhile, carboxylate groups can form complexation reactions with metal ions such as Ca^2+^ and Mg^2+^ on the surface of fractured rock masses, effectively enhancing the chemical bonding strength between the gel and rock matrix.

Excessively high SA dosage (AM:SA < 4:1) introduces a large number of anionic groups into the system, which reduces the crosslinking density and loosens the three-dimensional network structure of the gel. This deterioration leads to decreased mechanical strength and thermal stability. After aging at 80 °C, the compressive strength retention rate is less than 60%, and the bonding strength decreases by more than 40%, making the plugging layer vulnerable to failure under drilling fluid scouring. In contrast, an excessively low SA dosage (AM:SA > 4:1) results in insufficient hydrophilic groups. The 24 h equilibrium swelling ratio in formation water with a salinity of 20,000 mg/L is only 118%, which fails to fully fill the microfracture network. In addition, the insufficient carboxylate content weakens the chemical complexation effect with rock masses, yielding a bonding strength lower than 0.3 MPa and failing to achieve wellbore stabilization via rock consolidation.

When the dosage of sodium acrylate is excessively low, the equilibrium swelling ratio of the gel in formation water with a salinity of 20,000 mg/L is only 120%, which cannot fully fill the micro-fracture network of fractured formations, leading to a poor lost-circulation-control effect. Meanwhile, the low content of carboxylate groups results in weak chemical adhesion to the rock mass, with a bonding strength of less than 0.3 MPa, which fails to achieve the wellbore-stabilization effect.

#### 2.2.2. Effect of Crosslinking Agent Dosage on Gel Properties

**Figure 6:**
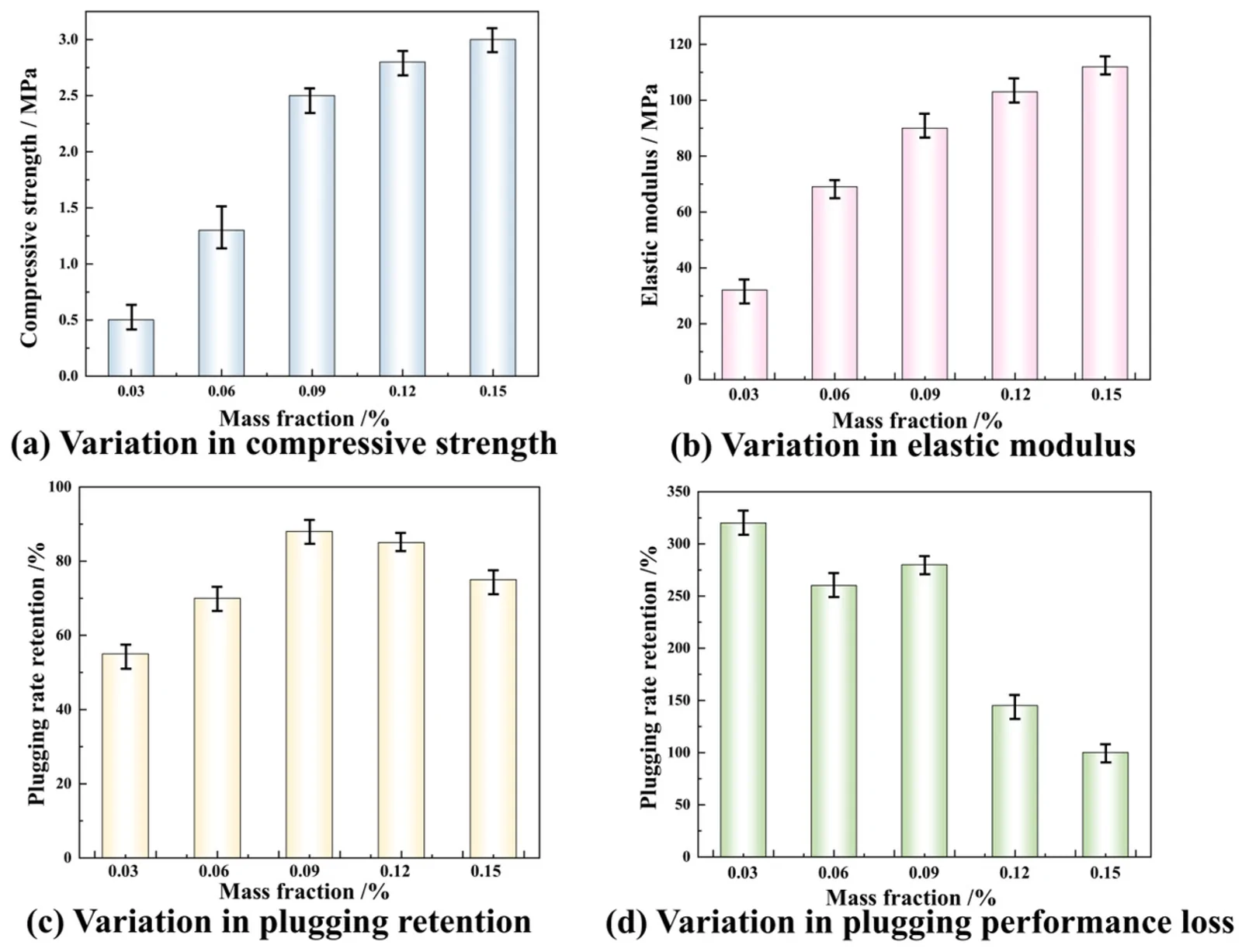
Effect of crosslinker dosage on gel properties. (**a**) Variation in compressive strength, (**b**) Variation in elastic modulus, (**c**) Variation in plugging retention, (**d**) Variation in plugging performance loss.

As shown in Figure 6, with other reaction conditions unchanged, the dosage of N,N′-methylenebisacrylamide (MBA) was adjusted ranging from 0.06% to 0.30% of the total system mass. The results indicate that the optimal MBA dosage is 0.09%. As a bifunctional crosslinker, MBA connects linear polymer chains into a three-dimensional network structure through polymerization reactions, and its dosage directly determines the crosslinking density of the gel.

An excessively high MBA dosage leads to an excessive crosslinking density and the restricted movement of molecular chain segments, which increases gel brittleness. The gel is prone to fracture under in situ stress, and its swelling performance is significantly reduced. On the contrary, an insufficient MBA dosage results in a low crosslinking density, which cannot support the formation of a complete three-dimensional network structure. The prepared gel possesses poor mechanical strength and is easily dispersed by the drilling fluid, resulting in the loss of plugging performance.

#### 2.2.3. Effect of Initiator Dosage on Gel Properties

**Figure 7:**
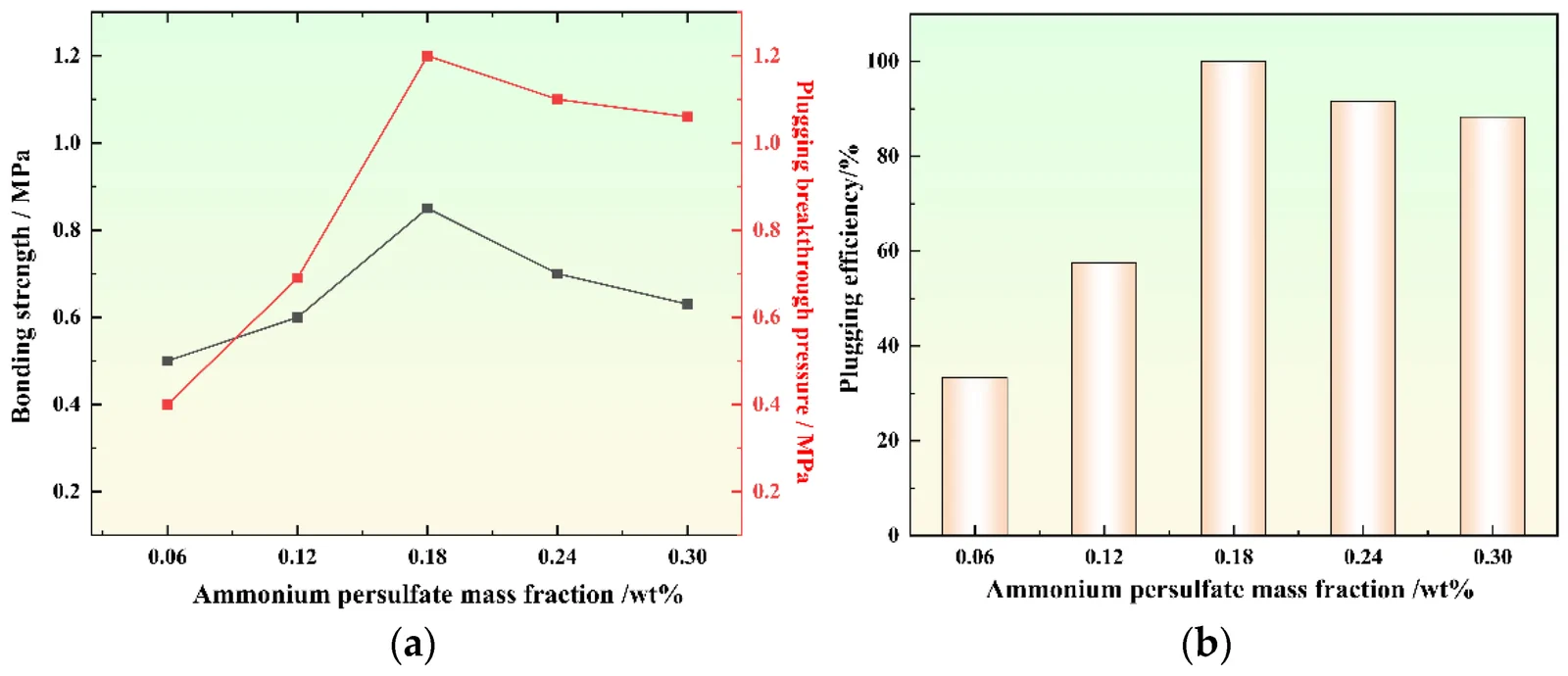
Effect of initiator dosage on gel properties. (**a**) Effect of initiator with different dosages on gel properties. (**b**) Effect of initiator with different dosages on sealing performance.

As shown in Figure 7, the dosage of the initiator affects the polymerization degree and structural properties of the gel by regulating the polymerization reaction rate and the generation amount of free radicals. When the dosage of APS is 0.18%, the gel is completely polymerized with the optimal comprehensive performance. When the initiator dosage is too low, the polymerization reaction rate is slow, the monomer polymerization is incomplete, and the gel has low strength and unstable performance. When the initiator dosage is too high, the polymerization reaction rate is too fast and a large number of free radicals are generated instantaneously in the system, which is prone to explosive polymerization. Bubbles and defects are easily generated inside the gel, resulting in a decrease in the structural compactness. Meanwhile, the molecular weight of the polymer decreases, leading to a significant reduction in the mechanical properties and thermal stability of the gel.

### 2.3. Effect of Reaction Temperature on Gel Properties

**Figure 8:**
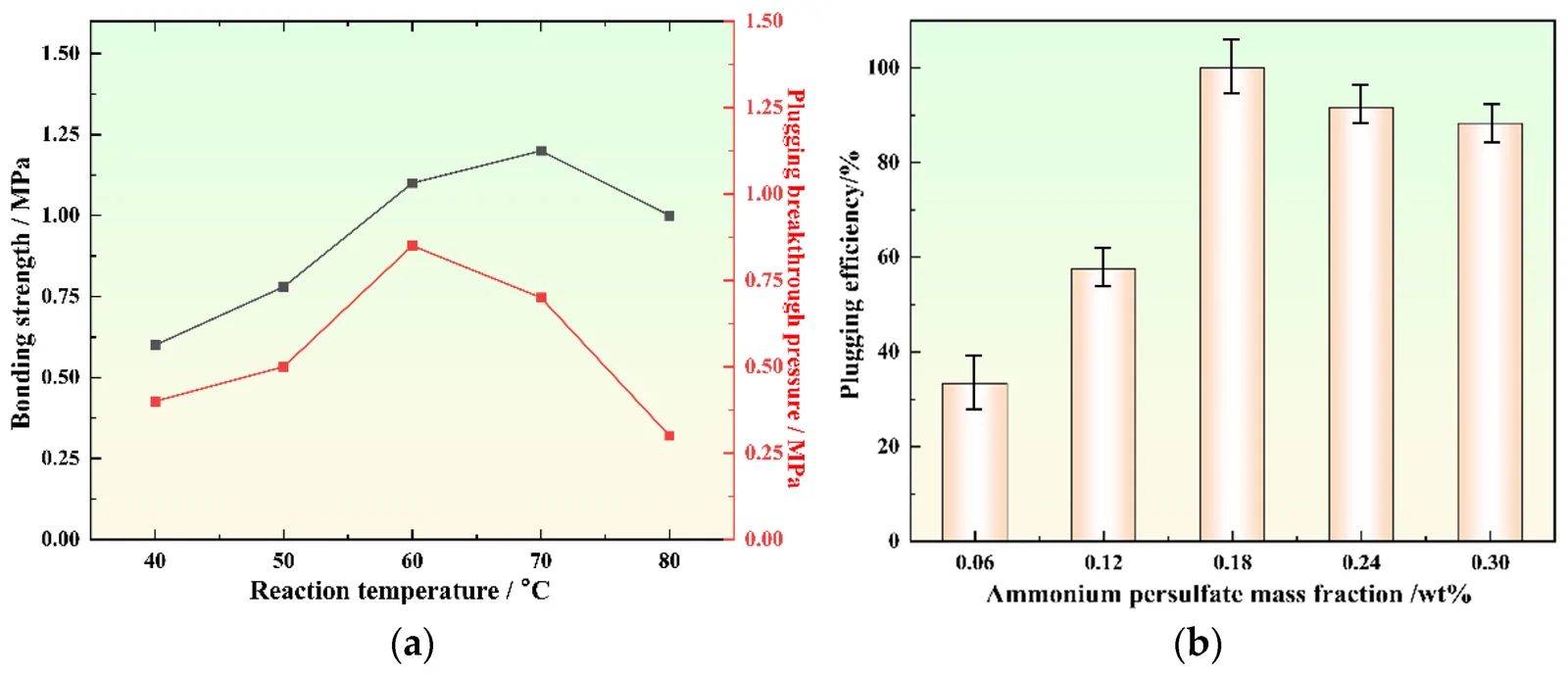
Effect of reaction temperature on gel properties. (**a**) Effect of different temperatures on gel properties. (**b**) Effect of different temperatures on sealing performance.

As shown in Figure 8, with other reaction conditions kept constant, the dosage of ammonium persulfate (APS) was adjusted from 0.06% to 0.30% of the total system mass, and the optimal APS dosage was determined to be 0.18%. APS initiates monomer polymerization by decomposing to produce sulfate radical ions. Its dosage regulates the polymerization rate and radical generation amount, thereby affecting the polymerization degree and structural properties of the gel.

An excessively high APS dosage accelerates the polymerization rate and generates a large number of free radicals instantaneously, which easily causes explosive polymerization. Numerous internal bubbles and structural defects are formed inside the gel, reducing structural compactness. Meanwhile, the molecular weight of the polymer decreases, leading to a significant deterioration in mechanical properties and thermal stability. In comparison, an insufficient APS dosage slows down the polymerization rate and results in incomplete monomer polymerization, producing gel materials with low strength and unstable performance.

The reaction temperature of 60 °C not only ensures the slow and uniform decomposition of the initiator, enables the continuous and stable generation of free radicals, and guarantees the complete polymerization of monomers and the formation of a uniform and dense three-dimensional network structure, but also effectively controls the polymerization reaction rate to avoid problems such as bubbles and molecular chain disorder caused by an excessively fast reaction. This endows the gel with excellent mechanical strength, elasticity, swelling properties, and bonding properties at the same time, which fully meets the requirements of lost-circulation control and wellbore stabilization in fractured formations.

### 2.4. Analysis of Core Performance Test Results of the Gel

#### 2.4.1. Swelling Property

**Figure 9:**
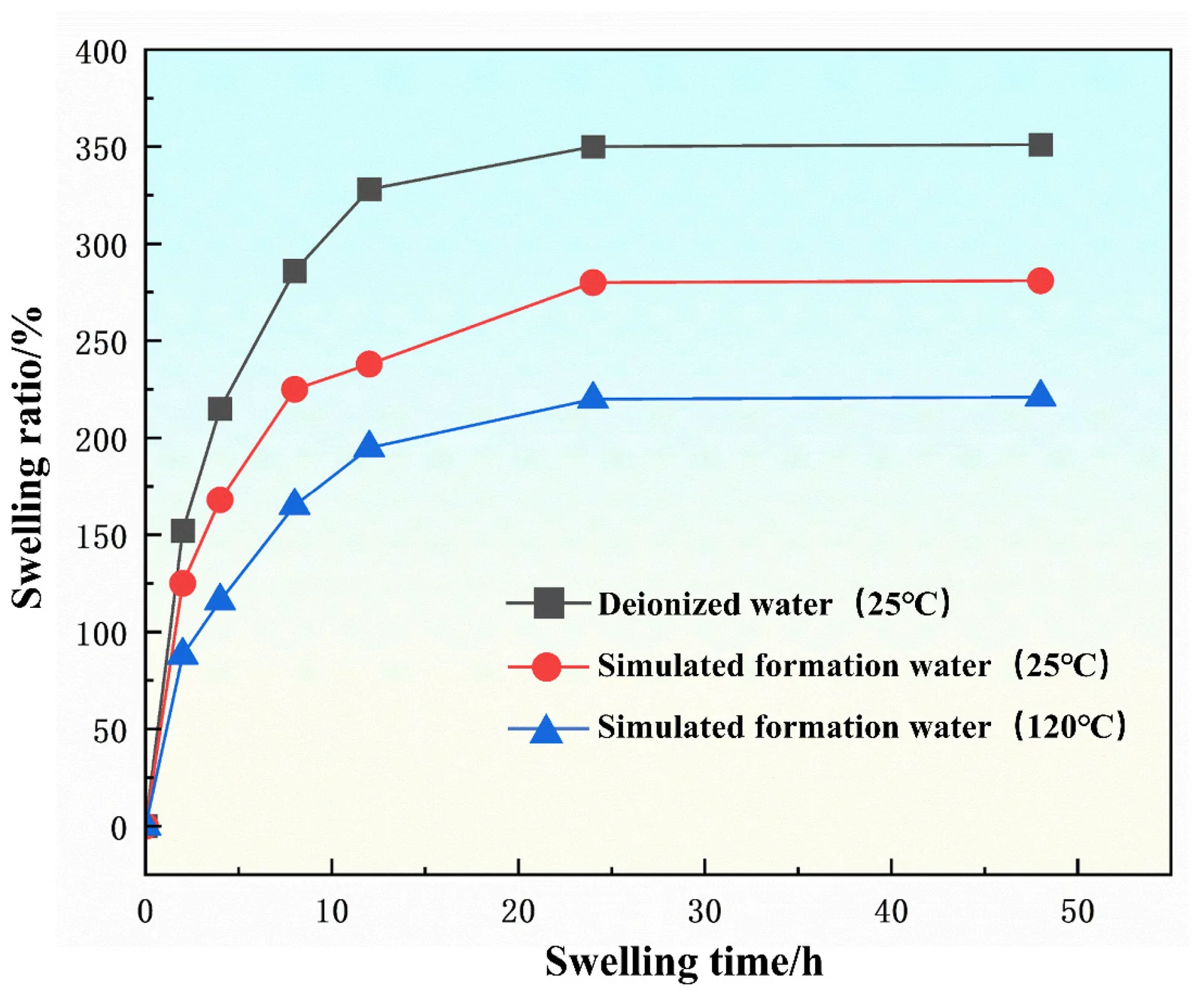
Test results of gel swelling property.

The swelling kinetic curves of the gel DF-PG in different media are shown in Figure 9. The test results show that the 24 h equilibrium swelling ratio of DF-PG in deionized water reaches 348%, and the swelling equilibrium is achieved at 48 h with no significant change in the swelling ratio. In simulated-formation water with a salinity of 20,000 mg/L, the 24 h equilibrium swelling ratio is 276%, the swelling ratio reaches 235% within 12 h, accounting for more than 85% of the total swelling ratio, and the swelling equilibrium is reached after 24 h.

This swelling characteristic is highly consistent with the engineering timeliness requirements of drilling in fractured formations. The rapid swelling within 12 h can fill the formation fracture channels in a short time to achieve rapid lost-circulation control. The swelling equilibrium reached at 24 h can avoid the volume-expansion stress caused by excessive swelling of the gel, and prevent secondary damage to the wellbore surrounding rock. Meanwhile, the gel presents moderate swelling and maintains a complete elastic structure after swelling without breakdown or dissolution phenomena, which can not only realize the full-domain filling of fractures, but also ensure the structural stability of the sealing layer. Under the high-temperature condition of 120 °C, the 24 h equilibrium swelling ratio of the gel in simulated-formation water can still reach 218%, and it maintains good elasticity and structural integrity after swelling, indicating that it has excellent high-temperature swelling stability and can adapt to the downhole environment of 80~120 °C.

Considering that sodium-acrylate-based hydrogels are susceptible to the ionic strength and divalent cations tend to cause the network shrinkage and syneresis of gels, two additional groups of simulated-formation water with total salinity of 10,000 mg/L and 30,000 mg/L were prepared with identical ion ratios to evaluate the salt tolerance and ionic stability of the DF-PG gel. The above simulated-formation water with different salinities contains typical monovalent and divalent cations existing in downhole environments, which were applied for gel swelling tests, high-temperature aging experiments, and core saturation treatment, respectively.

In simulated-formation water, salinity and divalent cations such as Ca^2+^ and Mg^2+^ exert remarkable effects on the swelling behavior of the gel. In standard simulated-formation water with a total salinity of 20,000 mg/L at 25 °C, the 24 h equilibrium swelling ratio of the gel reaches 276%, and more than 85% of the total swelling capacity is achieved within 12 h. When the salinity decreases to 10,000 mg/L and increases to 30,000 mg/L, the 24 h equilibrium swelling ratio changes to 301% and 242%, correspondingly. As the ionic strength rises, the negative charges on sodium-acrylate-modified molecular chains are shielded by surrounding cations, which reduces the electrostatic repulsion between molecular chains and leads to a slight decline in the swelling capacity of the gel.

**Figure 10:**
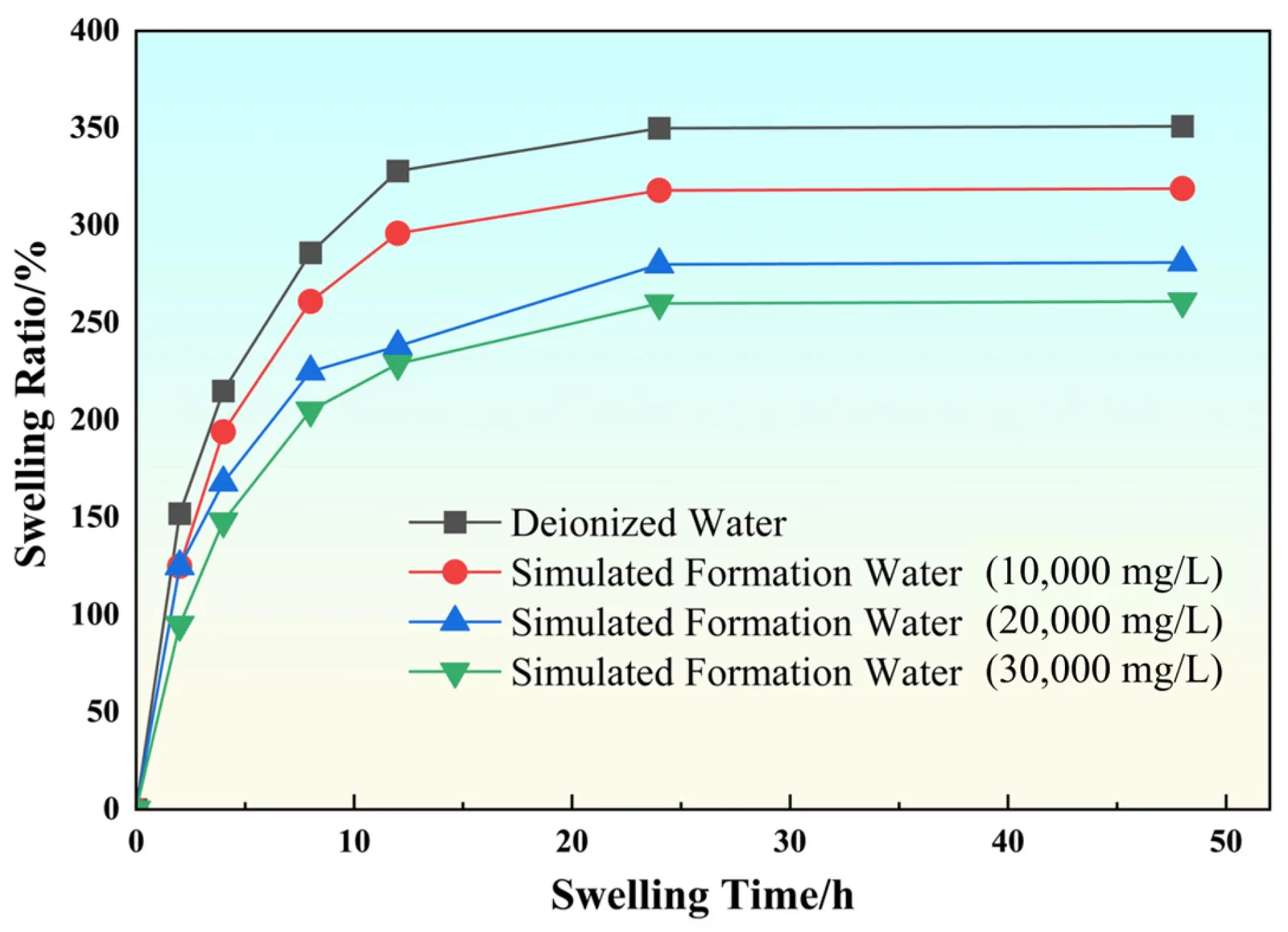
Fitting parameters of swelling kinetics and water diffusion coefficients of DF-PG gel.

Although the molecular chains of the gel contain sodium acrylate units, no obvious volume shrinkage or syneresis is observed across the salinity range of 10,000–30,000 mg/L. The covalently crosslinked network constructed by N,N′-methylenebisacrylamide effectively restricts the ionic crosslinking effect induced by Ca^2+^ and Mg^2+^ and prevents the severe coiling of polymer chains. The gel can maintain an intact elastic structure after swelling. When tested in standard simulated-formation water with a salinity of 20,000 mg/L at 120 °C, the 24 h equilibrium swelling ratio still reaches 218%. The gel retains a complete structure without dissolution or cracking after swelling. As shown in Figure 10.

The swelling kinetic data of DF-PG in different media and temperatures were fitted using the Korsmeyer–Peppas equation, and the fitting parameters and water diffusion coefficients are shown in Table 2. All fitting results have a goodness of fit *R*^2^ > 0.995, indicating that the Korsmeyer–Peppas equation can accurately describe the swelling process of the DF-PG gel.

The diffusion exponent n of DF-PG in all test conditions is between 0.45 and 0.89, indicating that the swelling process of the gel is dominated by non-Fickian diffusion. This is because the swelling of the acrylamide-based gel is a coupling process of water molecule diffusion and polymer network chain relaxation: the hydrophilic groups (amide group, carboxylate group) on the polymer chain rapidly combine with water molecules through hydrogen bonding, causing the polymer chain to stretch and the three-dimensional network to expand; at the same time, the crosslinked network structure limits the free relaxation of the polymer chain, making the water diffusion rate and polymer chain relaxation rate match each other, which is consistent with the structural characteristics of DF-PG with moderate crosslinking density.

Compared with deionized water, the diffusion exponent n and water diffusion coefficient D of DF-PG in simulated-formation water decrease slightly. This is due to the presence of high-valent cations (Ca^2+^, Mg^2+^) in the formation water, which will undergo electrostatic shielding with the anionic carboxylate groups on the polymer chain, reducing the electrostatic repulsion between polymer chains and inhibiting the expansion of the three-dimensional network, thus slowing down the water diffusion rate. However, the decrease amplitude is less than 25%, indicating that DF-PG has good salt resistance.

When the temperature rises from 25 °C to 120 °C, the water diffusion coefficient D increases by 75.6%, and the diffusion exponent n also increases slightly. This is because the increase in temperature enhances the thermal motion of water molecules and polymer chains, accelerating the water diffusion rate and the relaxation rate of polymer chains. Meanwhile, the hydrogen bond interaction between water molecules and hydrophilic groups is weakened at high temperature, which further promotes the penetration of water into the gel network. This characteristic ensures that DF-PG can still achieve rapid swelling and the filling of fractures in the high-temperature downhole environment.

#### 2.4.2. Temperature Resistance

**Figure 11:**
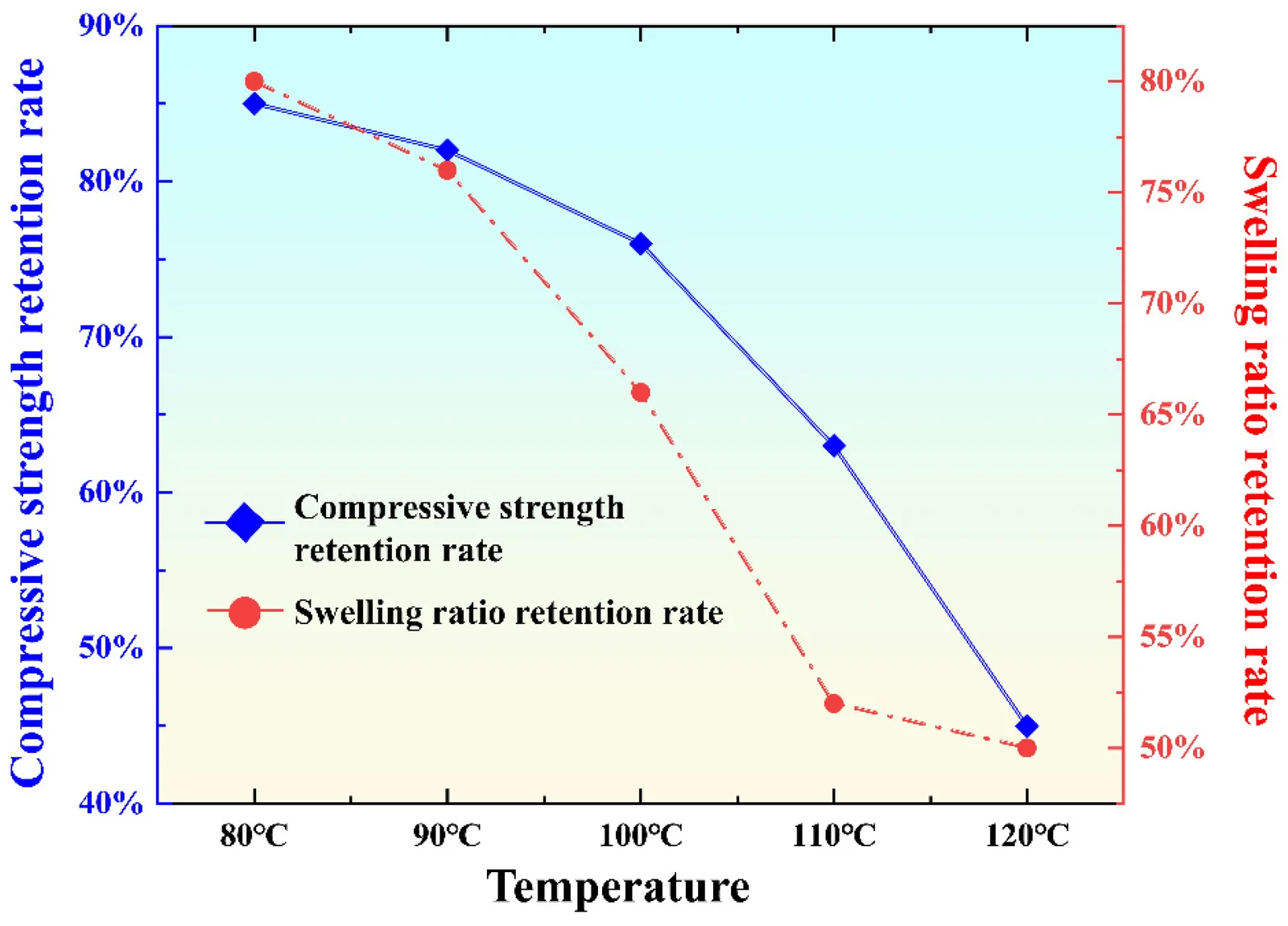
Test results of gel temperature resistance.

As shown in Figure 11, the aging performance test results show that after aging at 60 °C, DF-PG has no cracking, breakdown, or dissolution in appearance, only slight volume shrinkage occurs, the retention rate of compressive strength reaches 85%, and the retention rate of the swelling ratio reaches 80%. After aging at 80 °C, the retention rate of compressive strength still reaches 75%, and the retention rate of the swelling ratio reaches 70%, which meets the temperature requirements of fractured formations. After aging at 120 °C, the gel shows slight cracking, and the performance retention rate drops below 50%, indicating that its temperature resistance limit is about 120 °C.

#### 2.4.3. Mechanical Strength Performance

**Figure 12:**
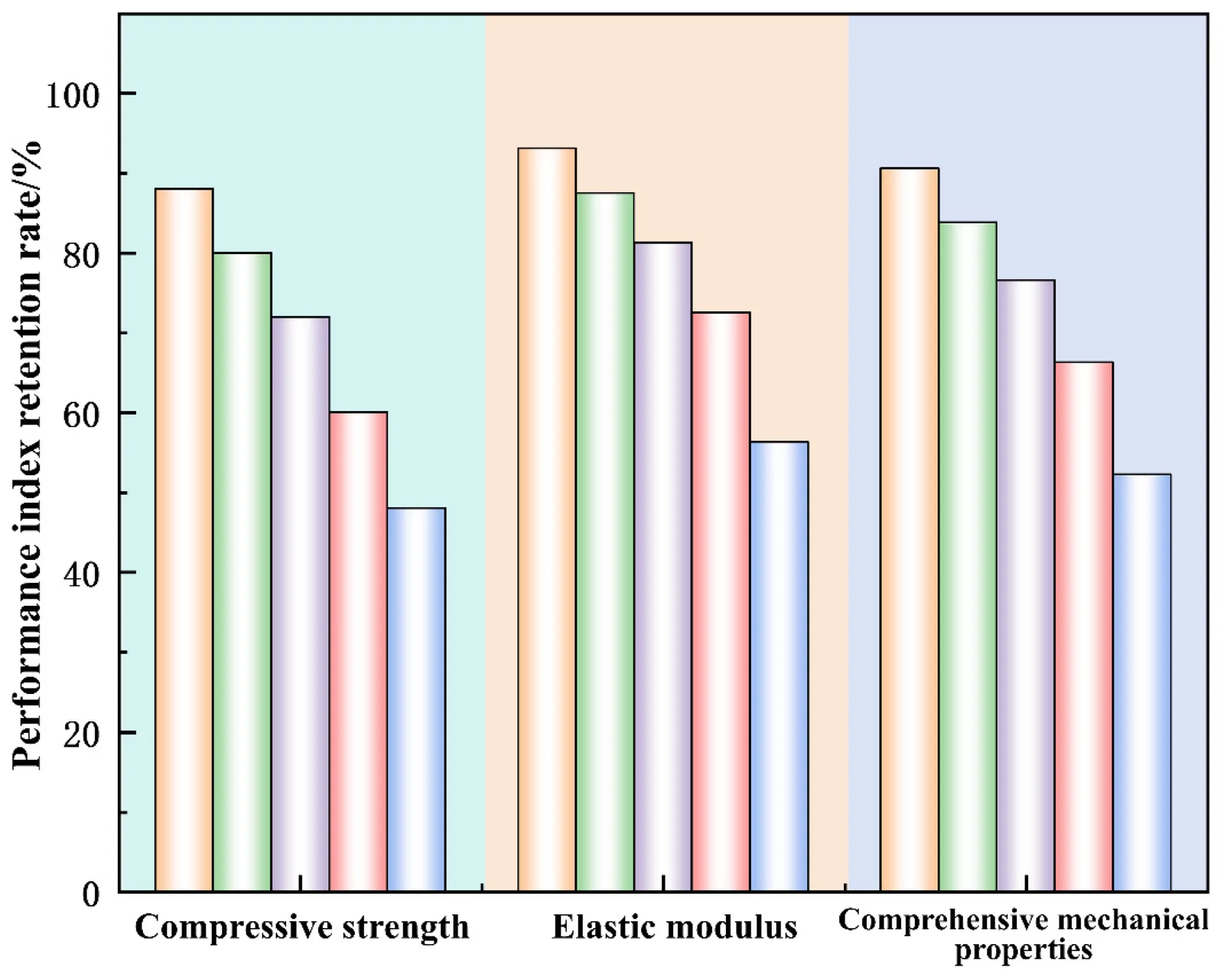
Test results of gel strength performance.

As shown in Figure 12, themechanical strength performance test results show that the gel prepared at room temperature has a compressive strength of 1.2 MPa, with excellent elasticity and compression resistance, which can resist the erosion effect of drilling fluid. After aging at 120 °C, the compressive strength of the gel still reaches 1.0 MPa, and the retention rate of mechanical properties is more than 70%, which can adapt to the in situ stress environment of fractured formations and avoid fracture failure of the sealing layer.

#### 2.4.4. Viscoelastic Properties

**Figure 13:**
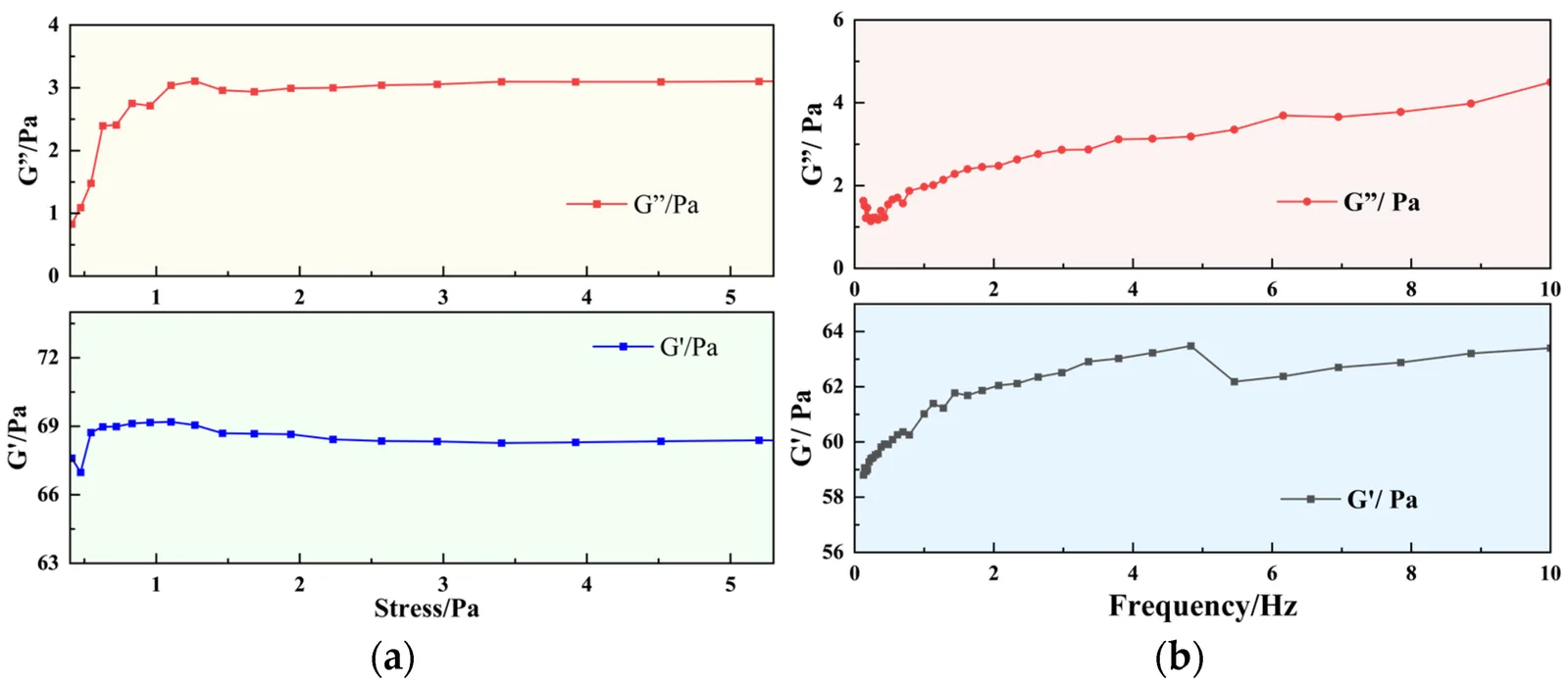
Viscoelastic characteristic curves of the gel. (**a**) Variation of storage modulus (G′) and loss modulus (G″) with shear stress. (**b**) Variation of storage modulus (G′) and loss modulus (G″) with frequency.

The curves of the storage modulus and loss modulus of the DF-PG gel versus stress and frequency are presented in Figure 13. The stress sweep results show that both G′ and G″ remain constant within the shear stress range of 0–1 Pa, which corresponds to the linear viscoelastic region of the gel. In this region, no irreversible damage occurs to the three-dimensional crosslinked network, and the mechanical response follows a linear relationship.

When the shear stress exceeds 1 Pa, G″ rises slightly and then stabilizes, while G′ decreases gradually. This indicates that minor elastic deformation emerges in the gel network, yet the overall structure remains intact. Throughout the entire tested stress range, G″ is always much higher than G″, and the loss tangent (tan δ = G″/G′) stays below 0.05. It demonstrates that the cured DF-PG gel exhibits prominent solid-like elastic characteristics. The dense and stable crosslinked network can effectively resist formation stress and dynamic shear from drilling fluid under downhole conditions.

The frequency sweep results (Figure 13b) reveal that both G′ and G″ increase gradually with the rise in frequency within the wide range of 0.1–10 Hz, and G′ remains more than one order of magnitude greater than G″. To be specific, G′ increases from 59 Pa to 64 Pa, and G″ rises from 1.2 Pa to 4.5 Pa. The loss tangent tan δ fluctuates slightly between 0.06 and 0.08. No obvious abrupt change occurs in the mechanical properties of the DF-PG gel in this frequency range, demonstrating that the gel is insensitive to the dynamic shear frequency. Hence, the sealing layer will not undergo structural damage or viscous flow under long-term scouring of the drilling fluid.

Furthermore, the extremely low tan δ value (<0.1) indicates that the gel has low energy dissipation and superior elastic recovery capability. Subjected to cyclic formation stress, it can maintain the integrity of the sealing structure and avoid sealing failure resulting from fatigue damage. It is worth noting that a minor step-like transition platform can be clearly observed on the storage modulus G′ curve of Figure 13b within the medium frequency range. This step feature is confirmed to be highly reproducible through three independent parallel tests using separately prepared DF-PG gel specimens. The frequency position corresponding to the step varies by less than ±0.1 Hz among replicates, and the G′ value at the step transition point has a relative deviation lower than 3%. This step signal originates from the reversible rearrangement of hydrogen-bonded aggregation domains inside the crosslinked network under dynamic shear, which is an intrinsic viscoelastic property of the AM/SA copolymer network, rather than instrument noise or experimental operation error. All oscillatory rheological tests in this paper were carried out in triplicate, and the displayed curves in Figure 13 represent the average value of three groups of data; tiny standard deviation error bars are covered by the curve lines due to small data dispersion.

#### 2.4.5. Sealing Performance

**Figure 14:**
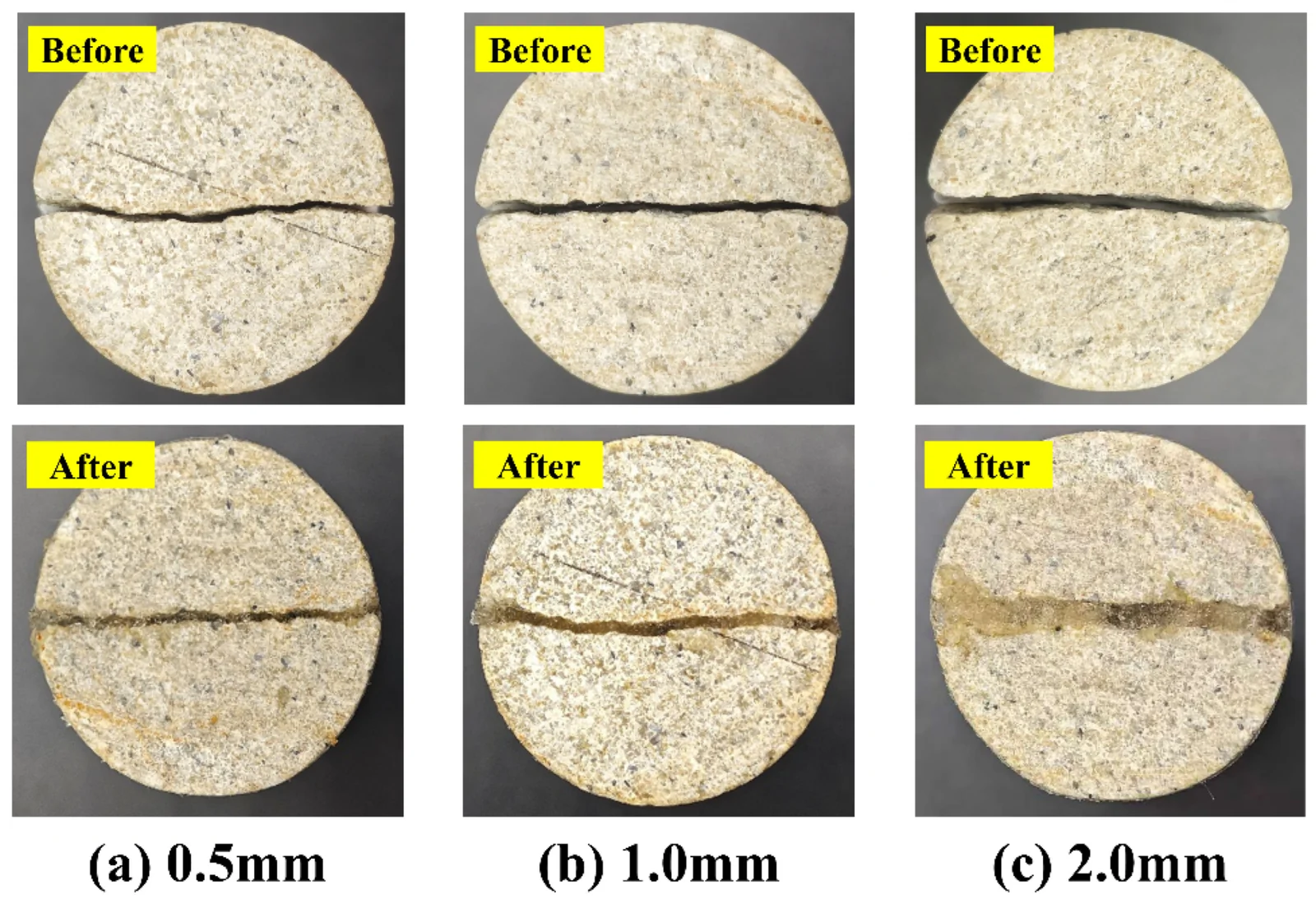
Plugging performance for fractures with different widths.

The test results show that DF-PG achieves a sealing efficiency of over 95% for fractured cores with fracture widths of 0.5~2.0 mm, with a breakthrough pressure of more than 1.2 MPa. For vuggy cores with a porosity of 20~30%, the sealing efficiency reaches above 90%, with a breakthrough pressure of over 0.8 MPa, demonstrating excellent full-domain sealing capacity. After 72 h of continuous scouring, the retention rate of sealing efficiency remains above 85%, indicating that the sealing layer has favorable anti-erosion performance and long-term stability,As shown in Figure 14.

Combined with the geometric parameters of prefabricated fractures, the influence of fracture characteristics on the breakthrough pressure was further analyzed. With the fracture length fixed at 50 mm and the fracture surface roughness (Ra) controlled within 18–22 μm, the breakthrough pressure of the gel-plugging layer gradually decreases as the fracture width increases. Specifically, the fractured core with a narrow fracture of 0.5 mm achieves the maximum breakthrough pressure of 1.45 MPa, the breakthrough pressure of the core with a medium fracture of 1.0 mm is 1.20 MPa, and the value drops to 1.05 MPa for the core with a wide fracture of 2.0 mm. This variation law is consistent with the fundamental principles of seepage mechanics: a larger fracture width leads to lower fluid flow resistance, and accordingly, the critical breakthrough pressure for failure of the plugging medium decreases. Meanwhile, the rough fracture surfaces can form mechanical interlocking with the elastic gel, which remarkably enhances the interfacial bonding strength between the gel and rock and prevents premature failure of the plugging layer along fracture surfaces.

**Figure 15:**
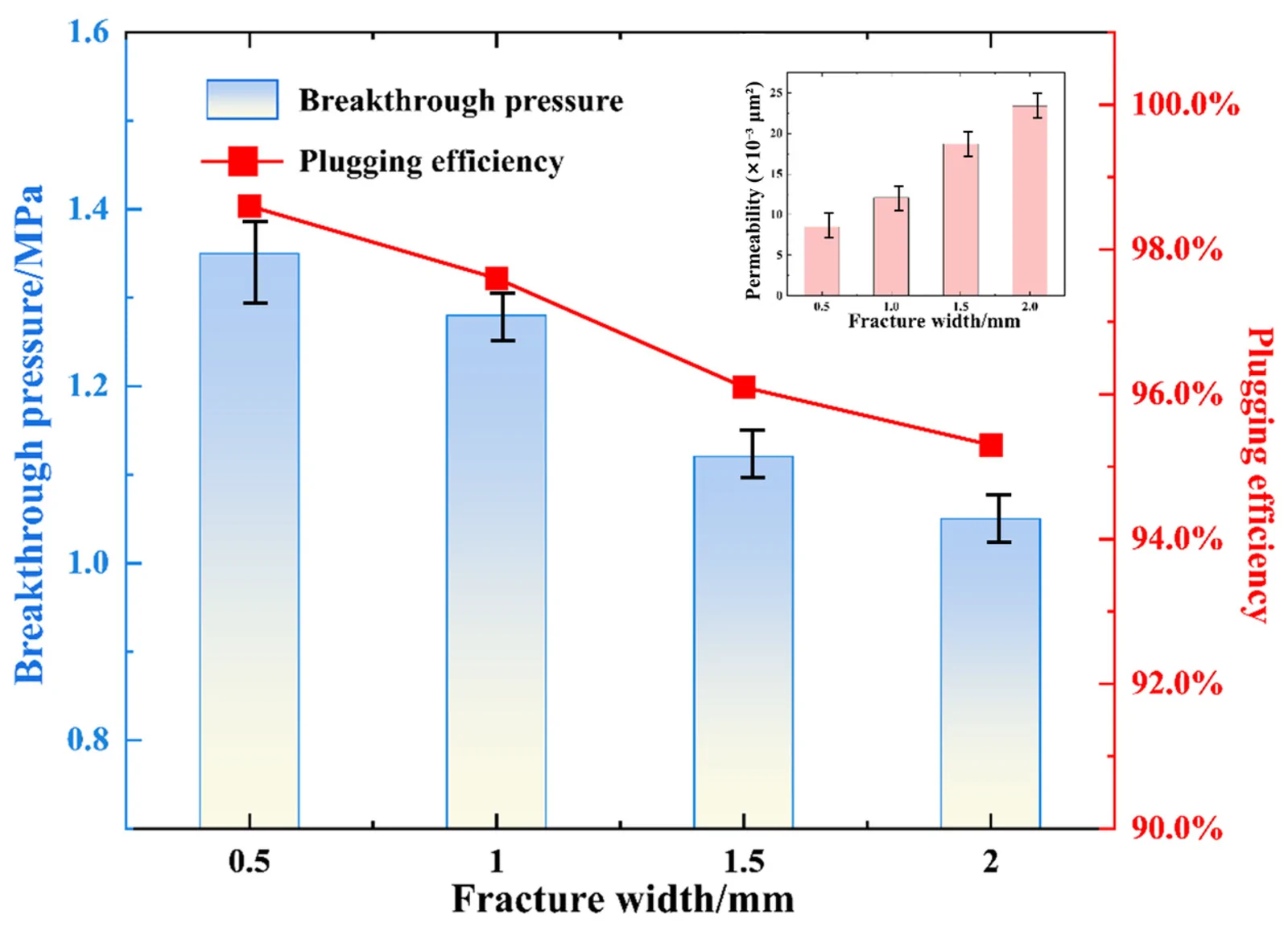
Sealing performance evaluation of the gel for fractures with different widths.

Based on the wellbore-strengthening hydrodynamic model, the fracture-closure stress theory, and the fluid–solid coupling theory proposed by Sarris and Gravanis [31,32], the actual downhole pressure difference in fractured formations is jointly determined by the hydrostatic pressure difference of the drilling fluid column, effective in situ stress of formations, and fracture closure stress. For conventional onshore fractured oil and gas fields in China, the operating downhole pressure difference ranges from 3.0 MPa to 12.0 MPa. The fracture closure stress is 2.5–8.0 MPa for shallow and medium-depth fractured reservoirs, while it can reach 8.0–18.0 MPa for deep-fractured formations. The core function of lost-circulation materials is to resist the combined pressure difference formed by the fluid seepage pressure and fracture closure stress, so as to ensure the long-term stability of the plugging layer. As shown in Figure 15.

**Figure 16:**
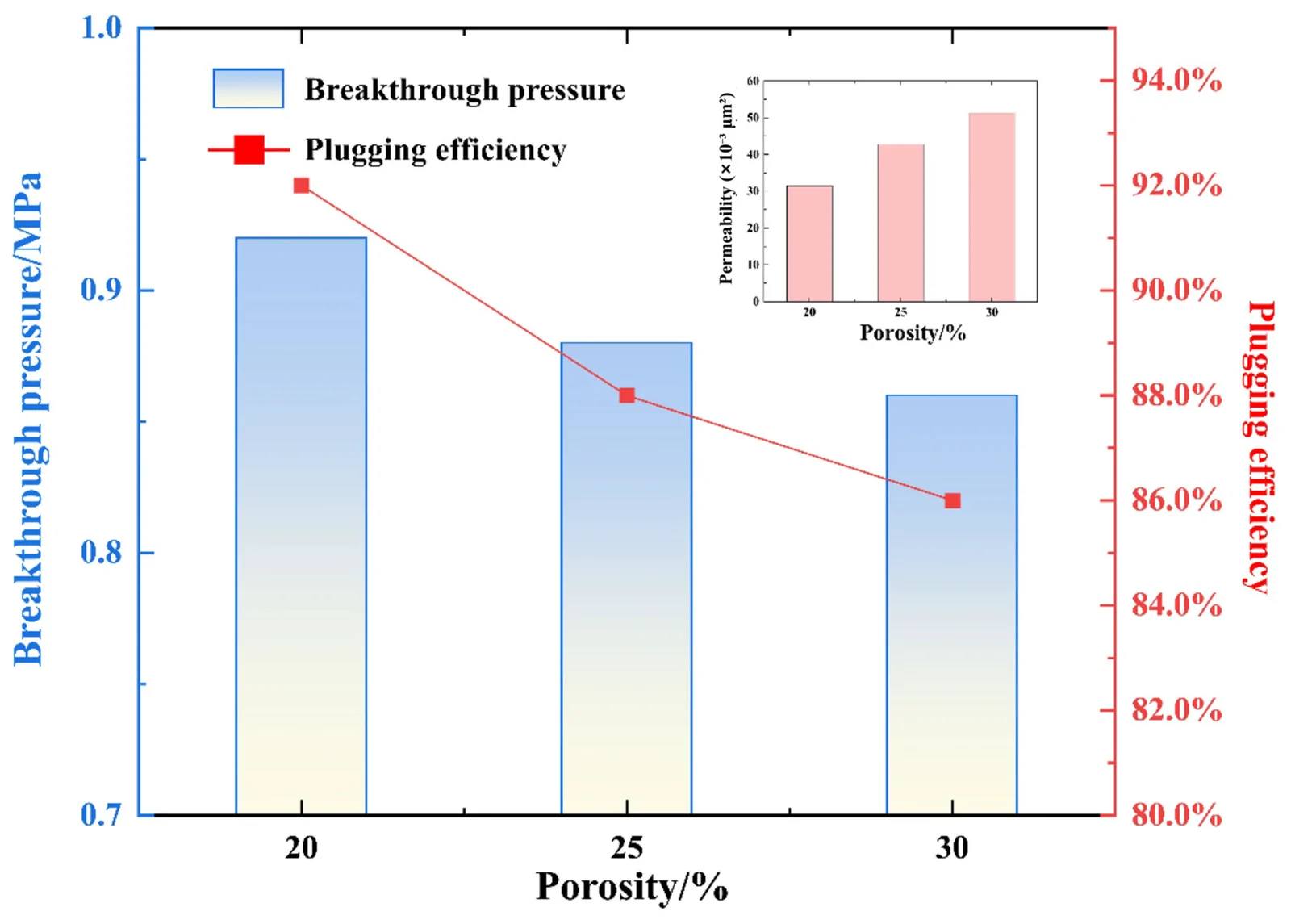
Sealing performance evaluation of the gel for cores with different porosities.

In accordance with the seepage similarity criterion and interfacial stress law, the scale-correction coefficient for breakthrough pressure was determined by integrating fracture geometric parameters and in situ stress conditions. In this laboratory test, the measured breakthrough pressure of the 1.0 mm-wide fracture is 1.2 MPa. After comprehensive correction considering the fracture length, in situ confining pressure, and superposition effect of fracture networks, the equivalent pressure-bearing capacity of the DF-PG gel-plugging layer under field-formation conditions can reach 3.2–7.5 MPa. As shown in Figure 16.

**Figure 17:**
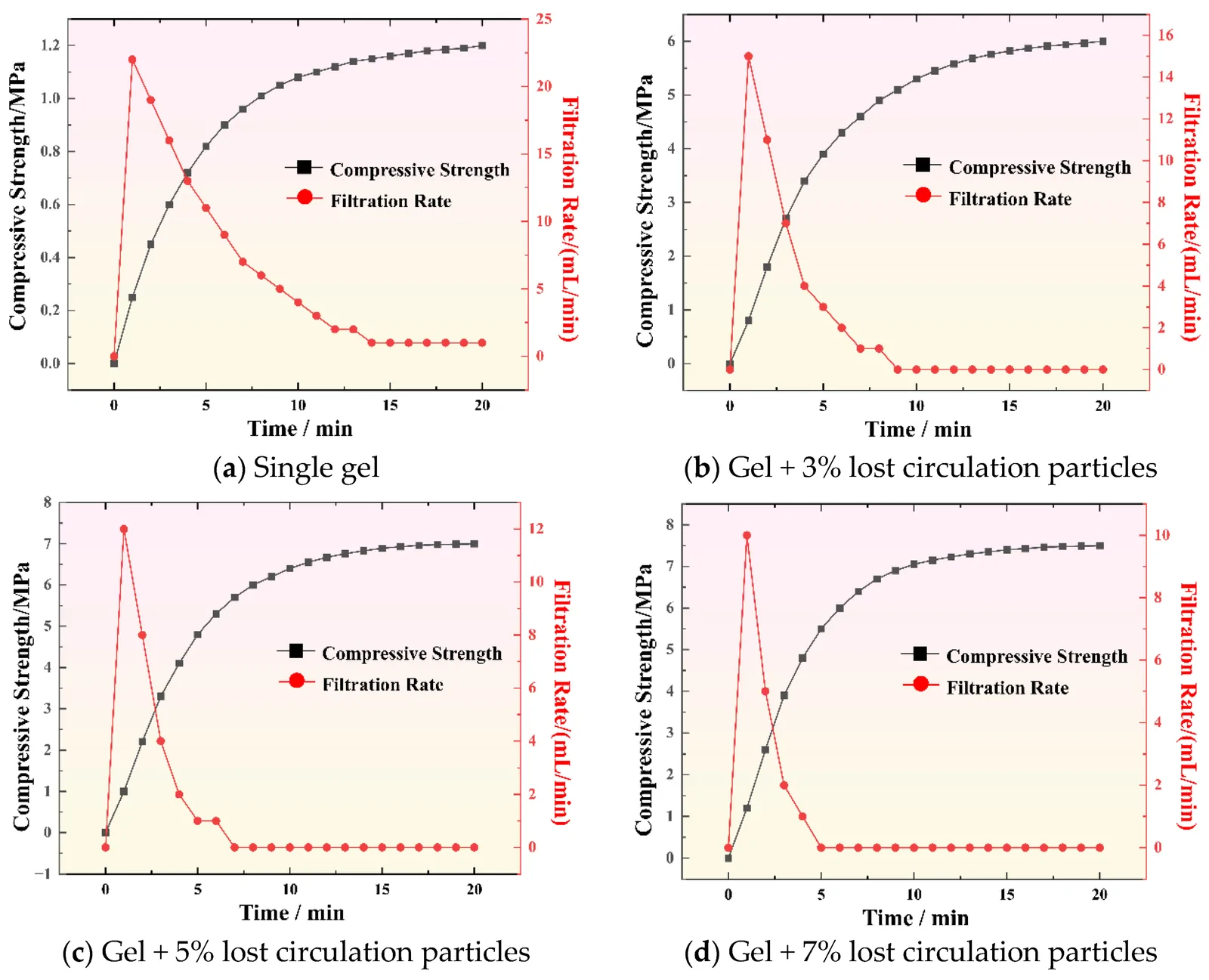
Lost circulation-control effect of the gel mixed with different proportions of lost-circulation particles.

The laboratory core data of breakthrough pressure and sealing efficiency provide quantitative support for DF-PG gel formula screening, yet there are inherent constraints when extrapolating small-scale experimental results to field-formation conditions. The scaling conversion is built on three idealized assumptions: geometric similarity, size-independent material properties, and equivalent confining pressure for subsurface stress. Meanwhile, the laboratory tests simplify real downhole conditions such as natural multi-branched fractures, the dynamic erosion of drilling fluid, and long-term stress fatigue. The boundary effect of small cores overestimates plugging pressure resistance, and the formation heterogeneity, together with dynamic loads from drill string vibration, further widens the deviation between laboratory test data and on-site actual plugging performance. As shown in Figure 17.

Therefore, laboratory breakthrough pressure is only applicable as a relative evaluation indicator for a horizontal comparison of material performance, and the absolute experimental values need to be corrected based on formation geological features, the in situ stress status, and the fracture-development scale before field deployment. Subsequent research will carry out large-scale physical simulation and multi-scale numerical simulation to construct a complete performance-scaling method, so as to further improve the guiding effect of experimental results on field lost circulation control engineering.

The sealing mechanism of the gel is an integrated “infiltration-swelling-filling “ process. After mixing with water, the dry gel powder penetrates into the multi-scale fractures and rock mass gaps of fractured formations in a low-viscosity fluid state, followed by rapid water absorption and swelling to self-adaptively fill the entire fracture network. Meanwhile, hydration crosslinking occurs to form a dense elastic sealing layer, blocking the lost-circulation channels. Through physical adhesion and chemical complexation with the rock mass surface, the gel cements loose rock fragments, enabling the sealing layer to form an integral whole with the formation surrounding rock. This avoids the inherent limitations of conventional bridging lost-circulation materials, including only surface bridging, the inability to fill micro-fractures, and easy shedding of the sealing layer.

#### 2.4.6. Compatibility with Drilling Fluid

**Figure 18:**
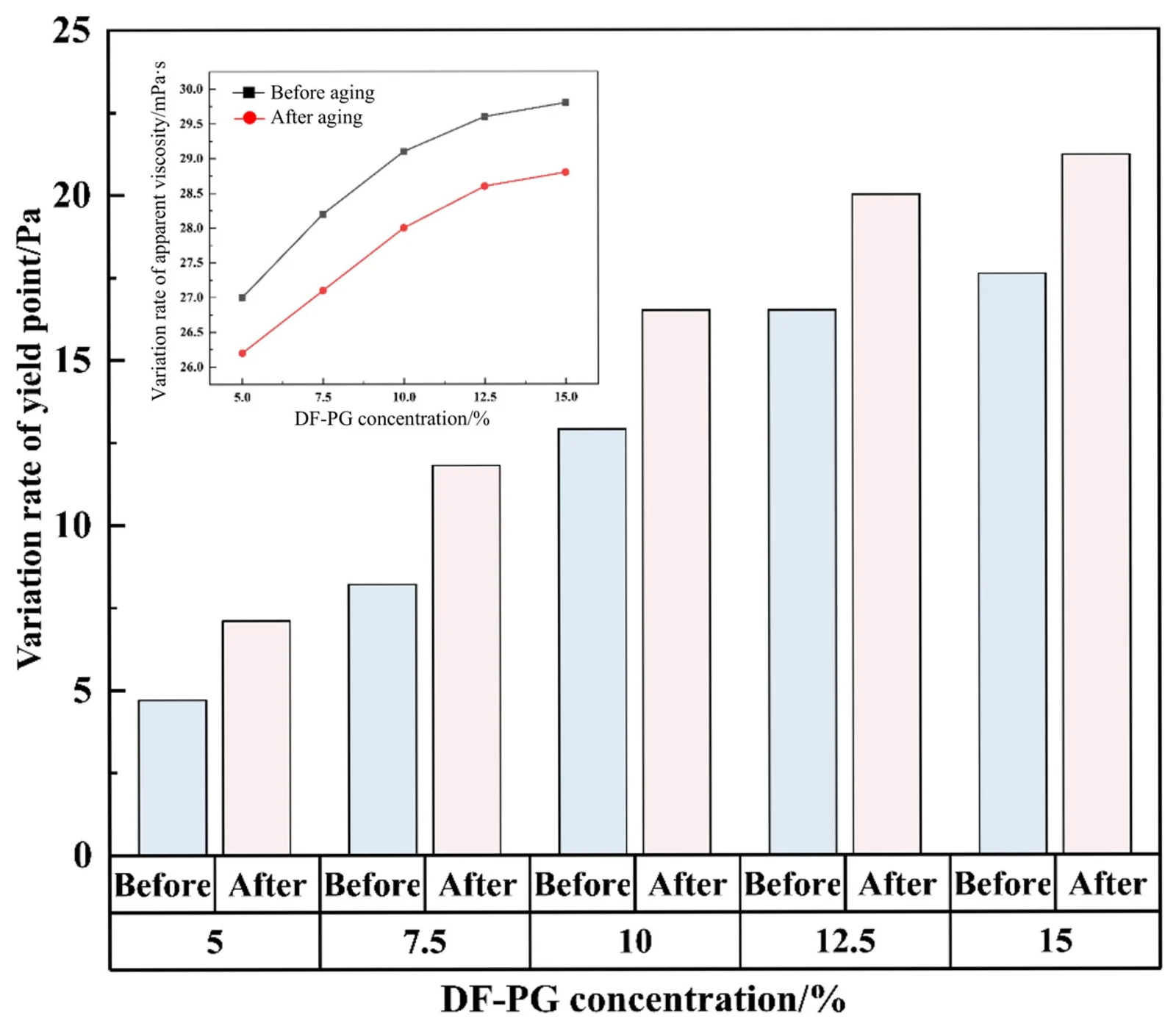
Compatibility evaluation of the gel with drilling fluid.

The compatibility test results with drilling fluid show that after adding DF-PG into the water-based drilling fluids commonly used in fractured formations at a ratio of 5~15%, the drilling fluids maintain a homogeneous appearance with no flocculation, sedimentation, delamination, or scum, presenting excellent dispersibility. At room temperature, the change rates of apparent viscosity (AV) and plastic viscosity (PV) of the drilling fluids in each experimental group are all ≤15%, and the change rate of the yield point (YP) is ≤18%. After aging at 120 °C for 16 h, the change rate of rheological parameters is still ≤20%, and the rheological properties of the drilling fluids remain stable, which meets the compatibility judgment criteria. This indicates that the gel has favorable compatibility with drilling fluids and can be directly compounded with drilling fluids for use, satisfying the requirements of field construction. As shown in Figure 18.

#### 2.4.7. Comparison with Conventional Guar-Gum-Based Lost-Circulation Material

As shown in Table 3, to further evaluate the plugging performance advantage of DF-PG, a comparative study was conducted using a conventional guar-gum-based lost-circulation material (GG-LCM), which is widely applied in field lost-circulation treatments because of its low cost and rapid hydration characteristics. The same testing procedures described in Section 2.4 were adopted for both materials under identical experimental conditions.

The comparison results indicated that DF-PG exhibited significantly superior overall performance compared with the guar gum system. The equilibrium swelling ratio of DF-PG reached 268%, whereas the guar gum material showed a swelling ratio of only 185%. The enhanced swelling capacity of DF-PG is attributed to the presence of hydrophilic amide and carboxylate groups, as well as its three-dimensional crosslinked network structure, which enables continuous water absorption and adaptive fracture filling.

In terms of thermal stability, the differences between the two materials became more pronounced after aging at 150 °C for 72 h. DF-PG maintained 82.4% of its original compressive strength, while the guar gum system retained only 51.7%. The thermal degradation of polysaccharide chains in guar gum resulted in a substantial reduction in structural integrity, whereas the covalently crosslinked AM-SA network of DF-PG remained relatively stable under high-temperature conditions.

Mechanical property testing further demonstrated the advantages of DF-PG. The compressive strength of DF-PG reached 1.26 MPa, compared with 0.73 MPa for the guar gum system. The higher mechanical strength enables the gel to withstand formation stress and drilling-fluid erosion more effectively, thereby improving the long-term stability of the sealing layer.

For fractured-core plugging tests, DF-PG achieved a plugging efficiency of 96.3% and a breakthrough pressure of 8.7 MPa, whereas the guar gum system achieved only an 84.5% plugging efficiency and 5.2 MPa breakthrough pressure. The superior sealing performance of DF-PG is mainly attributed to its elastic three-dimensional network structure, which can penetrate into fracture networks, establish strong interfacial adhesion with the rock surface, and maintain sealing integrity during pressure fluctuations.

These results confirm that DF-PG provides significant improvements in swelling capability, thermal resistance, mechanical strength, and plugging performance compared with conventional guar-gum-based lost-circulation materials. Therefore, DF-PG exhibits greater potential for lost-circulation control and wellbore stabilization in fractured formations.

### 2.5. Feasibility Analysis for Field Application

The DF-PG gel exhibits excellent comprehensive performance under laboratory conditions. Its feasibility for field application is elaborated as follows:

Pumpability: The base solution of the gel possesses favorable fluidity before gelation. Its viscosity ranges from 12 to 15 mPa·s at 25 °C, which is close to that of conventional water-based drilling fluids. Therefore, it can be directly pumped by existing drilling pumps without additional equipment modification.

Gelation time regulation: By adjusting the dosage of initiator APS (0.12–0.24%) or adding an appropriate amount of retarder such as sodium citrate, the gelation time can be flexibly controlled within 30–120 min, meeting the pumping and construction requirements under different well depths and temperature conditions.

Long-term durability: After aging for 30 days at 120 °C and 20 MPa, the retention rate of compressive strength of the DF-PG gel remains 55%, and the retention rate of the plugging efficiency reaches 72%. It can satisfy the engineering requirements for short-term plugging and wellbore stabilization in fractured formations.

Economy: All raw materials are bulk industrial chemicals with low cost. The preparation process is simple, and the gel can be directly formulated at on-site fluid-mixing stations. Its overall cost is lower than that of conventional chemical lost-circulation materials.

Meanwhile, this system has certain application limitations. Firstly, its temperature-resistance limit is approximately 120 °C, so it is not applicable to ultra-deep wells with formation temperatures exceeding 120 °C. Secondly, in sulfur-rich formations, hydrogen sulfide (H_2_S) may damage the amide groups and crosslinking bonds of the gel and degrade its performance. Further molecular modification is required to improve its sulfur resistance. Field pilot tests will be carried out in the follow-up work to optimize the on-site construction process and verify its practical application performance.

## 3. Conclusions

Aiming at the geological and engineering characteristics of fractured formations, including fragmented rock mass, network-distributed fractures, poor cementation, and the readily occurred wellbore instability and severe lost circulation, which form a vicious circle during drilling operations, this paper prepared an acrylamide-based lost-circulation-control gel DF-PG adapted to such formations via aqueous-solution free radical polymerization, using acrylamide (AM) and sodium acrylate (AANa) as polymerizable monomers, N,N′-methylenebisacrylamide (MBA) as the crosslinking agent, and ammonium persulfate (APS) as the initiator. Through systematic optimization of the mass fraction of reaction components and reaction process parameters, the structural characteristics, performance-regulation law, and formation adaptability of the gel were clarified, and multi-dimensional core performance tests were completed. The main conclusions are as follows:

(1) In this paper, the integrated gel DF-PG for lost-circulation control and wellbore stabilization was prepared via aqueous-solution free radical polymerization using AM and AANa as copolymerization monomers. The optimal preparation formula and process were determined through single-factor experiments: 84.5% deionized water, 6.8% AM, 1.7% AANa (AM:AANa mass ratio of 4:1), 0.09% MBA, 0.18% APS, and a constant-temperature reaction at 60 °C for 4 h. Under this formula, the gel is completely polymerized and forms a dense and continuous three-dimensional network structure, balancing the mechanical strength, swelling property, bonding performance, and thermal stability.

(2) DF-PG has excellent comprehensive properties adapted to fractured formations. The compressive strength at room temperature reaches 1.2 MPa, the retention rate of mechanical properties after aging at 120 °C for 16 h reaches 62%, and the temperature-resistance limit is about 120 °C. In simulated-formation water with a salinity of 20,000 mg/L, the 24 h equilibrium swelling ratio reaches 276%, more than 85% of the total swelling capacity is completed within 12 h, and the performance is stable when the salinity is ≤30,000 mg/L, showing excellent salt resistance. For fractured cores with fracture widths of 0.5~2.0 mm, the sealing efficiency is ≥95% and the breakthrough pressure is ≥1.2 MPa; for vuggy cores with a porosity of 20~30%, the sealing efficiency is ≥90%, and the retention rate of sealing efficiency is ≥85% after 72 h of scouring. It has favorable compatibility with field commonly used water-based drilling fluids at a dosage of 5~15% and can be directly compounded for field application.

(3) DF-PG can realize the dual functions of lost-circulation control and wellbore stabilization through the integrated “infiltration-swelling-filling “ mechanism. In a low-viscosity fluid state, it can penetrate into the multi-scale fracture network, swell rapidly to self-adaptively fill the lost-circulation channels, and form a dense elastic sealing layer after gelation. Meanwhile, it can cement loose rock fragments through physical adhesion and chemical complexation, increasing the compressive strength of the fractured rock mass by more than 4 times and fundamentally solving the problem of wellbore instability. The composite system with 6% dosage of rigid particles can significantly improve the pressure-bearing capacity for large-fracture sealing, which meets the treatment requirements of severe lost circulation in fractured formations.

(4) The DF-PG gel prepared in this paper adapts to the engineering and geological characteristics of fractured formations and has excellent injectability, sealing performance, and rock-mass-cementation capacity, with strong feasibility for field application. It can provide a new technical scheme and experimental support for lost-circulation control and wellbore-stability management in drilling operations of fractured formations. Future work will further optimize the delayed crosslinking performance and high temperature resistance of the gel and expand its application in the high-temperature fractured formations of ultra-deep wells.

## 4. Materials and Methods

### 4.1. Experimental Materials

Acrylamide (AM, analytical reagent, purity ≥ 99%) and sodium acrylate (SA, analytical reagent, purity ≥ 98%) were adopted as polymerization monomers. Sodium acrylate can be either prepared using neutralizing acrylic acid or directly used as a solid reagent. N,N′-methylenebisacrylamide (MBA, analytical reagent, purity ≥ 98%) was used as the crosslinker, and ammonium persulfate (APS, analytical reagent, purity ≥ 98%) served as the initiator. Laboratory-prepared deionized water was applied throughout the experiments.

As shown in Table 4, the simulated fractured formation water was formulated according to the salinity of on-site formation water, which contained ions such as Na^+^, Ca^2+^, and Mg^2+^, with a total salinity of 20,000 mg/L. The specific ion contents are listed in the table below.

The characterization and test instruments used were as follows: Fourier Transform Infrared Spectrometer (FT-IR, spectral range 4000~400 cm^−1^), Universal Electronic Testing Machine (accuracy 0.01 MPa), Constant Temperature Blast Drying Oven (temperature control accuracy ±1 °C), Freeze Vacuum Dryer (vacuum degree ≤ 10 Pa), High Temperature and High Pressure (HTHP) Consistometer (maximum temperature 200 °C, maximum pressure 50 MPa), Artificial Core Sealing Evaluation Device (maximum pressure 30 MPa), Six-Speed Rotational Viscometer (0~600 r/min), Analytical Electronic Balance (accuracy 0.0001 g), Magnetic Stirrer (rotational speed 0~2000 r/min), High Temperature and High Pressure (HTHP) Reactor (maximum temperature 200 °C, maximum pressure 30 MPa), 100-mesh standard sieve, 250 mL Erlenmeyer flasks, glass petri dishes, filter paper, etc.

### 4.2. Preparation Method of the Gel

**Figure 19:**
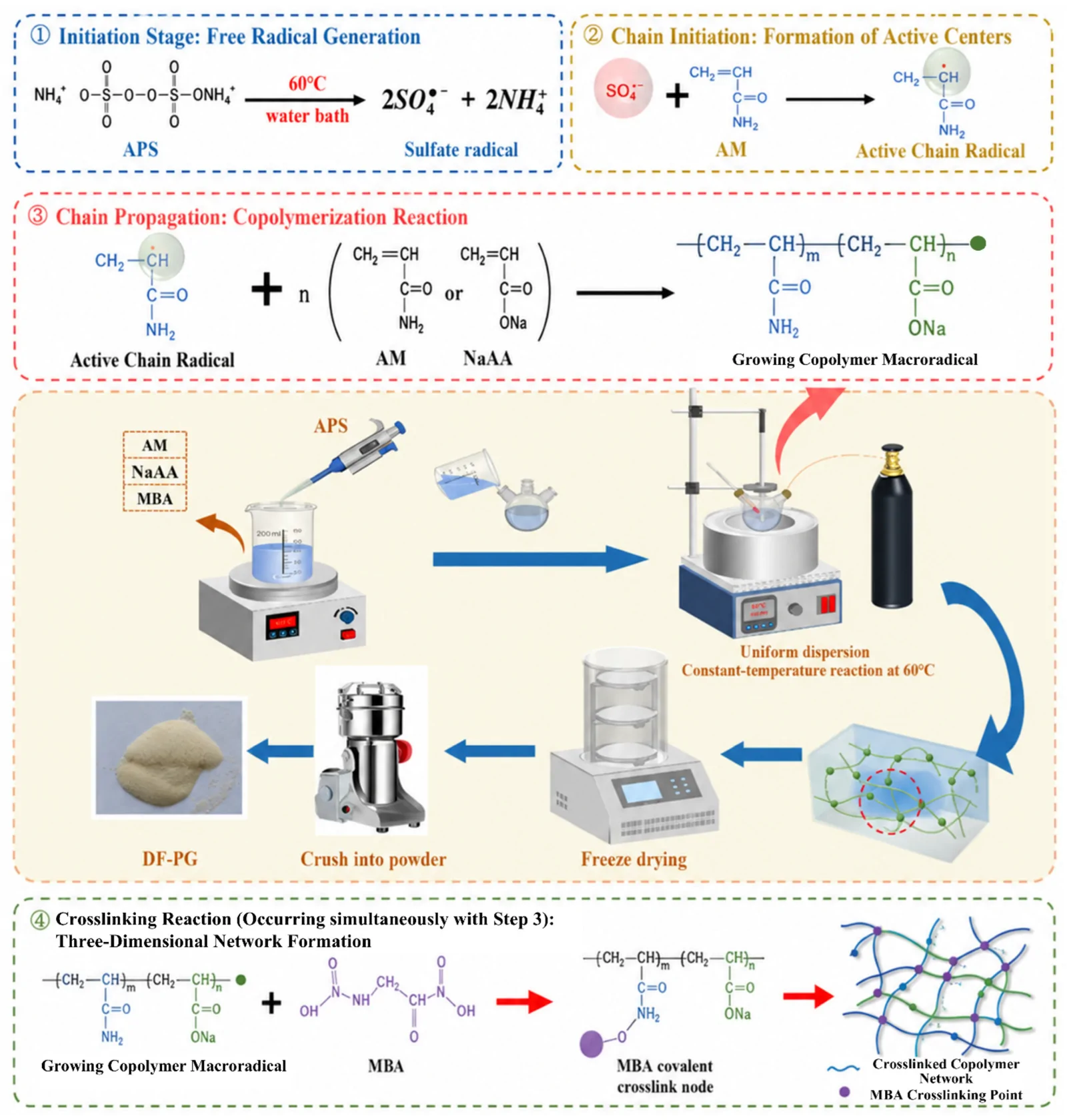
Preparation process of gel DF-PG.

As shown in Figure 19, the lost-circulation-control gel DF-PG for fractured formations was prepared via aqueous-solution free radical polymerization, with the specific process as follows: deionized water was used as the reaction medium, accounting for 84.5% of the total mass of the 50 g standard reaction system, corresponding to 42.25 g (approximately 42.25 mL, density ≈ 1.00 g/mL) of deionized water. This volume of deionized water was transferred into a 100 mL beaker, which was then placed on a magnetic stirrer with stirring started at a rotational speed of 800 r/min. First, 3.4 g acrylamide (6.8% of total mass, molar concentration 0.95 mol/L) was slowly added, and stirring was continued for 15 min until it was completely dissolved to form a homogeneous aqueous solution. Maintaining the above stirring rate, 0.85 g sodium acrylate (1.7% of total mass, molar concentration 0.20 mol/L) was slowly added into the system, and stirring was continued for 10 min until it was completely dissolved to ensure uniform mixing of the monomers. Subsequently, 0.045 g N,N′-methylenebisacrylamide (MBA, 0.09% of total mass, molar concentration 5.8 mmol/L) was added as the crosslinking agent, and stirring was continued for 5 min at the constant stirring rate to completely dissolve and uniformly disperse the crosslinking agent. Finally, 0.09 g of ammonium persulfate (APS, 0.18% of total mass, molar concentration 7.9 mmol/L) was added as the initiator.

After stirring for 3 min until the system was homogeneous, the stirring was stopped to complete the preparation of the reaction system. The beaker containing the prepared reaction system was placed in a constant-temperature water bath reaction device, the temperature was set at 60 °C, the beaker mouth was sealed, and the constant-temperature reaction was carried out for 4 h until a transparent or translucent elastic colloid was formed in the system. After the reaction was completed, the gel product was taken out, cut into small pieces, placed in a glass petri dish, and washed repeatedly with anhydrous ethanol 3 to 4 times, with each soaking time of 10 min, to remove unreacted monomers and residual aqueous solution. The washed gel was placed in a freeze vacuum dryer and dried at −50 °C and 5 Pa for 24 h. After being taken out, the gel was ground into powder with an agate mortar, sieved through a 100-mesh standard sieve, and then loaded into a sealed bag and placed in a desiccator for later use.

### 4.3. Gel Characterization Methods

Chemical Structure Characterization: The dried gel powder was mixed with KBr at a mass ratio of 1:100, fully ground, and pressed into a pellet. The pellet was placed in the sample chamber of a Fourier Transform Infrared Spectrometer (FT-IR) for infrared spectrum scanning, with a scanning range of 4000~400 cm^−1^, 32 scans, and a resolution of 4 cm^−1^. Through the change of absorption peaks of characteristic functional groups, the degree of completion of the polymerization reaction and the formation of the crosslinked network were analyzed, and the molecular structure characteristics of the gel were clarified [33].

Apparent Performance Characterization: An optical microscope (magnification: 10~40×) was used to observe the microscopic morphology of the gel. Meanwhile, the transparency and homogeneity of the fresh gel were observed by naked eyes, and defects such as bubbles and cracks were recorded to evaluate the apparent performance of the gel [34].

### 4.4. Gel Performance Test Methods

Combined with the geological characteristics of fractured formations, including the multi-scale fractures, loose rock mass, poor cementation, and strong scouring effect of drilling fluid, a performance test system for the lost-circulation-control gel DF-PG was established from six core dimensions: swelling property, temperature resistance, mechanical strength, sealing performance, salt resistance, and compatibility with drilling fluid. The operation procedures, instrument parameters, and calculation methods of each test item were refined [35]. For each group of experiments, 3 to 5 parallel samples were set up, and the average value after removing outliers was taken as the test result. The details are as follows:

#### 4.4.1. Swelling Property Test

The gravimetric method was adopted to test the swelling kinetic characteristics and equilibrium swelling ratio of the DF-PG gel in different media at different temperatures, with the specific operation as follows: the dried gel powder sieved through a 100-mesh screen was accurately weighed into equal-mass samples and placed in a number of 250 mL Erlenmeyer flasks, respectively; 100 mL of deionized water and simulated fractured formation water were added into each flask separately. After sealing the bottle mouths, the Erlenmeyer flasks were placed in a constant-temperature blast drying oven at 25 °C and 60 °C and a nitrogen-filled 150 °C High Temperature and High Pressure (HTHP) reactor at 20 MPa, respectively, for constant temperature swelling. Sampling was conducted at different time nodes of 2 h, 4 h, 8 h, 12 h, 24 h, and 48 h during swelling. After quickly absorbing the free moisture on the sample surface with qualitative filter paper, the swollen mass of the gel was immediately weighed, and the data were recorded. After sampling, the sample was returned to the original flask to continue sealed swelling. When the sample reached swelling equilibrium after 48 h of swelling, its final swollen mass was recorded. The swelling ratio of the gel was calculated according to Equation (1), and the swelling law of the gel in different media at different temperatures was analyzed.

(1) $$\gamma =\frac{m_{t}-m_{0}}{m_{0}}\times 100\%$$

where γ is the swelling ratio of the gel, %; $m_{t}$ is the mass of the gel after swelling for time, g; and $m_{0}$ is the initial mass of the gel before drying, g.

#### 4.4.2. Temperature Resistance Test

The temperature resistance of the gel was evaluated based on dual indicators of the performance-retention rate after high-temperature aging and high-temperature gelation stability, to match the wellbore temperature characteristics during drilling in fractured formations (conventional well temperature 80~160 °C).

For the high-temperature aging performance test, cube specimens with uniform specifications and defect-free surfaces were prepared from the freshly prepared elastic gel. After being equally divided into multiple groups, the compressive strength of the specimens at room temperature was measured, and the initial data were recorded. Each group of specimens was placed in a High Temperature and High Pressure (HTHP) reactor filled with simulated-formation water respectively. After nitrogen was injected to 20 MPa, the specimens were aged at a constant temperature and pressure for 72 h at 120 °C, 140 °C, 160 °C, and 180 °C, respectively. After aging, the reactor was cooled and depressurized, the specimens were taken out to observe the change in apparent morphology, and the compressive strength and swelling ratio after aging were measured. The performance-retention rate was calculated according to Equation (2), to evaluate the performance stability of the gel after high-temperature aging [36].

(2) $$\eta =\frac{X_{1}}{X_{0}}\times 100\%$$

where η is the performance retention rate, %; X_1_ is the performance index of the gel after high-temperature aging (compressive strength/swelling ratio); and X_0_ is the initial performance index of the gel before high-temperature aging (compressive strength/swelling ratio).

For the high-temperature gelation stability test, multiple portions of the reaction system without the initiator were prepared according to the gel-preparation method. After the initiator ammonium persulfate (APS) was added and stirred evenly, the system was quickly placed in a HTHP Consistometer, and the test was carried out under simulated formation temperature and pressure conditions of 150 °C and 30 MPa. The initial gelation time and complete gelation time of the system were observed and recorded, and the viscosity change was recorded at regular intervals during the gelation process. After the system was completely gelled, curing was continued at a constant temperature and pressure for 4 h. The gel was taken out to observe the gelation state, and its gelation stability under high temperature and high pressure was evaluated according to whether there were phenomena such as delamination, breakdown, and excessive bubbles.

#### 4.4.3. Mechanical Strength Performance Test

The compressive strength and elastic modulus of the gel were tested using a Universal Electronic Testing Machine, to evaluate its resistance to formation stress and drilling fluid erosion. The freshly prepared elastic gel was processed into standard cube specimens with uniform sizes and defect-free surfaces. The Universal Electronic Testing Machine was zeroed and set to uniaxial compression test mode, with a loading rate of 1 mm/min, and specimen fracture was taken as the test-termination condition [15].

The specimen was smoothly placed on the loading platform of the testing machine to ensure it was centered without eccentric load. The testing machine was started to carry out the compression test, and the load–strain data were recorded in real time. The maximum compressive load was recorded after the specimen fractured. All specimens were tested according to the above procedures. After removing the maximum and minimum values, the average value of 8 specimens was taken as the final test result. Meanwhile, the compressive strength was calculated according to Equation (3), the load–strain curve was converted into a stress–strain curve, and the elastic modulus was calculated according to Equation (4).

(3) $$\sigma =\frac{F_{max}}{S}$$

where σ is the compressive strength of the gel, MPa; *F_max_* is the maximum compressive load, N; and S is the compressed area of the specimen, mm^2^.

(4) $$E=\frac{∆\sigma }{∆\varepsilon }$$

where E is the elastic modulus of the gel, MPa; ∆σ is the stress variation, MPa; and ∆ε is the strain variation.

After parallel tests were conducted on multiple specimens, the final test result was determined as the average value after eliminating extreme values. Meanwhile, the specimens were subjected to high-temperature and high-pressure aging at 150 °C and 20 MPa for 72 h, and the above test steps were repeated to measure the mechanical strength indexes of the gel after aging.

#### 4.4.4. Oscillatory Rheological Property Test

A rotational rheometer equipped with a 25 mm parallel plate fixture and a plate gap of 1 mm was used to quantitatively characterize the dynamic viscoelasticity of DF-PG gel. All tests were conducted at a constant temperature of 30 °C to simulate the ambient-temperature environment of normal-temperature intervals in downhole formations.

Stress sweep test: The oscillation frequency was fixed at 1 Hz, and the shear stress was set to sweep from 0 Pa to 5 Pa. The variations in the storage modulus (G′) and loss modulus (G″) with shear stress were continuously measured to determine the linear viscoelastic region (LVR) and critical yield stress for the fluid–solid transition of the gel.

Frequency sweep test: Based on the stress sweep results, a constant shear strain within the linear viscoelastic region was selected. The oscillation frequency was scanned from 0.05 Hz to 10 Hz. The relationships of G′, G″, and the loss tangent (tanδ = G″/G′) versus frequency were measured to analyze the frequency dependence of viscoelasticity and the shear-response behavior of the gel.

#### 4.4.5. Sealing Performance Test

**Figure 20:**
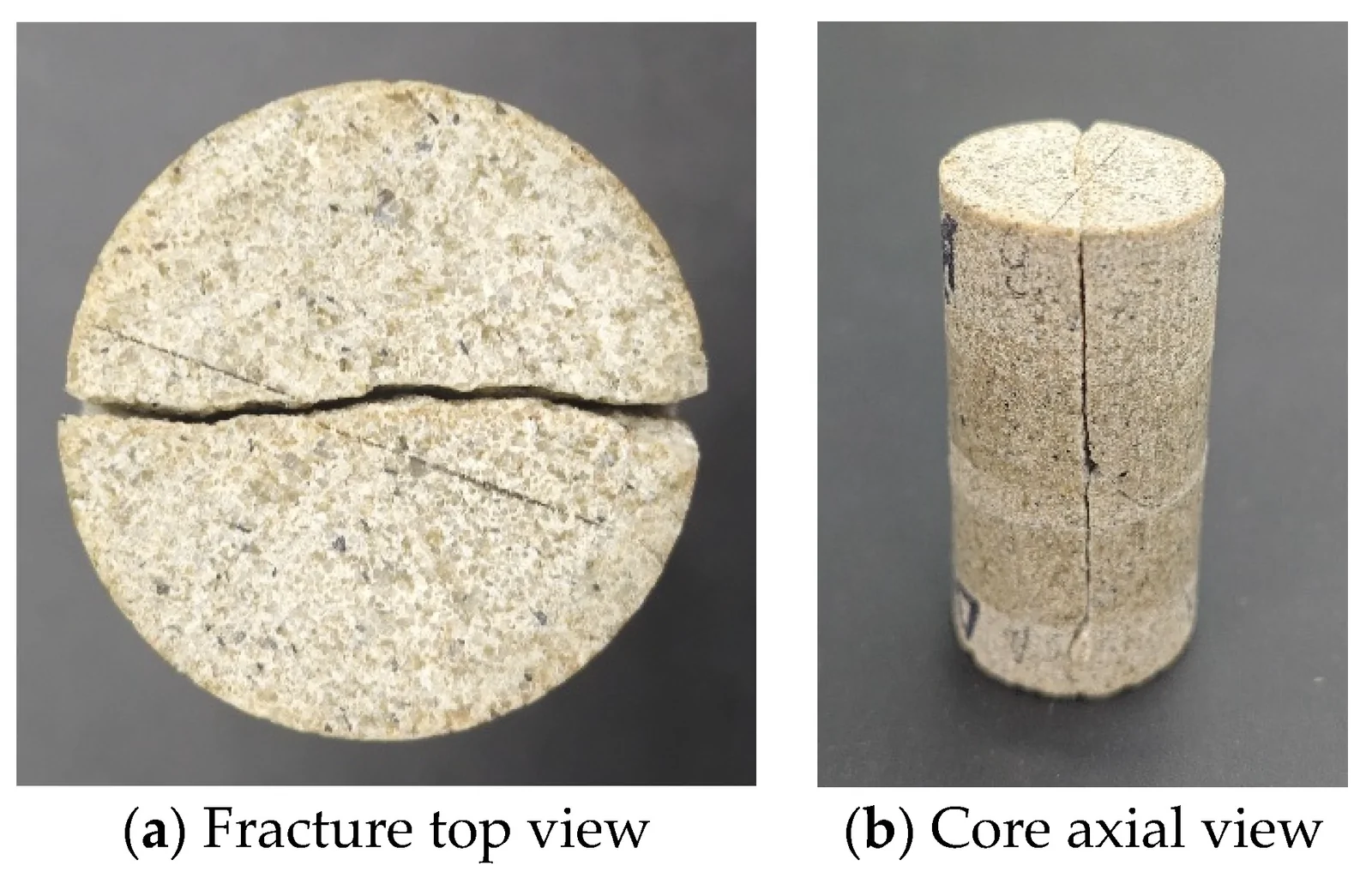
Initial state of the core.

As shown in Figure 20, an artificial fractured core sealing-evaluation device was used to simulate the multi-scale fracture lost-circulation characteristics of fractured formations, to test the sealing efficiency, breakthrough pressure, and sealing-layer stability of the gel and simultaneously evaluate the cementation and strengthening effect of the sealing layer on the fractured core [37].

Artificial cores simulating fractured formations with the specification of φ25 mm × 50 mm were prepared, including fractured cores with fracture widths of 0.5 mm, 1.0 mm, and 2.0 mm and vuggy cores with a porosity of 20~30%. All cores were vacuum dried at 60 °C for 24 h, vacuum pumped for 4 h, and then saturated with simulated-formation water for 24 h, and the initial permeability of the cores K_0_ was tested and recorded.

The lost-circulation slurry was prepared according to the mass ratio of the gel powder to water-based drilling fluid of 10:90 and stirred at a high speed of 3000 r/min for 20 min until the system was homogeneous. The saturated artificial core was loaded into the core holder of the device, and nitrogen was injected into the annulus to 10 MPa to prevent channeling and leakage. The lost-circulation slurry was injected into the core through a high-pressure injection pump at a constant rate of 0.5 mL/min and an injection pressure of 5 MPa, until the slurry completely filled the fractures and vugs of the core.

After the injection was completed, the valves at both ends of the core were closed, and curing was carried out at 25 °C with an annulus pressure of 10 MPa for 24 h, to allow the gel to fully swell and crosslink in the fractures and vugs of the core to form a sealing layer. After curing, simulated-formation water was injected into the core at a rate of 0.2 mL/min with a stepwise pressure increase. After the pressure was stabilized, the permeability after sealing K_1_ was tested, and the sealing efficiency was calculated according to Equation (4).

The pressure was continuously increased stepwise by 0.5 MPa and maintained for 5 min at each pressure level. When the sudden pressure drop was ≥30%, the pressure at this time was recorded as the breakthrough pressure of the gel. Subsequently, the injection pressure was set to 80% of the breakthrough pressure, and simulated-formation water was continuously injected into the core for scouring for 72 h. The permeability after scouring K_2_ was tested, and the retention rate of sealing efficiency was calculated according to Equation (5), to evaluate the anti-erosion stability of the sealing layer [38].

(5) $$\varphi =\frac{K_{0}-K_{1}}{K_{0}}\;\times \;100\%$$

where φ is the sealing efficiency of the gel, %; K_0_ is the initial permeability of the core, mD; and *K*_1_ is the permeability of the core after sealing, mD.

(6) $$\varphi^{\prime }=\frac{K_{0}-K_{2}}{K_{0}}\;\times \;100\%$$

where φ′ is the retention rate of gel sealing efficiency, %, and K_2_ is the permeability of the core after erosion, mD.

#### 4.4.6. Compatibility Test with Drilling Fluid

Based on the water-based drilling fluids commonly used in fractured formations (polysulfonate drilling fluid, potassium-based polymer drilling fluid), the compatibility between the gel and drilling fluids was tested, with the requirements of no flocculation or sedimentation and no significant alteration in the rheological properties and wellbore-inhibition capacity of the drilling fluids. The specific procedures are as follows:

The water-based drilling fluid was equally divided into multiple portions, one of which was used as the blank group, and the dried gel powder was added to the rest at the mass ratios of 5%, 10%, and 15%, respectively. All samples were stirred at a high speed of 3000 r/min for 20 min to form a homogeneous mixed system and left to stand for 2 h after stirring. The appearance of the drilling fluid was observed by naked eyes, and the compatibility was preliminarily judged according to the presence of flocculation, sedimentation, delamination, scum, and other phenomena. A six-speed rotational viscometer was used to measure the full-range rotational speed readings of the drilling fluids in the blank group and each experimental group at room temperature, and the rheological parameters, including the apparent viscosity (AV), plastic viscosity (PV), and yield point (YP), were calculated according to Equations (7)–(9).

(7) $$AV\left(mPa\cdot s\right)\;=\;1/2\;\times \;600\;r/min$$

(8) PV(mPa·s) = 600 r/min − 300 r/min

(9) $$YP\left(Pa\right)\;=\;AV-PV$$

Subsequently, all drilling fluid samples were sealed in high-temperature aging cans and placed in a constant-temperature forced-air drying oven at 150 °C for 16 h of aging. After aging, the samples were naturally cooled to room temperature and stirred thoroughly, and the above rheological parameter test procedures were repeated. The change rate of rheological parameters was calculated according to Equation (10). The compatibility between the gel and drilling fluid was comprehensively evaluated based on the visual observation results and the parameter change rates.

(10) $$\omega \;=\;\frac{Y_{1}\;-\;Y_{0}}{Y_{0}}\times 100\%$$

where ω is the change rate of rheological parameters, %; Y_1_ is the rheological parameter (AV/PV/YP) of the drilling fluid in the experimental group; and Y_0_ is the rheological parameter (AV/PV/YP) of the drilling fluid in the blank group.

The compatibility criteria were as follows: no flocculation or sedimentation in the drilling fluid, and the change rates of apparent viscosity and plastic viscosity were both ≤20%.

#### 4.4.7. Thermogravimetric Analysis Test

A simultaneous thermal analyzer was used to quantitatively characterize the thermal stability of the gel DF-PG and clarify the thermal decomposition behavior and temperature resistance limit of the material. The whole test was carried out under a high-purity nitrogen atmosphere with a nitrogen flow rate set at 50 mL/min. The sample was the fresh gel prepared with the optimal formula, which was freeze-vacuum dried for 48 h to a constant weight and then ground into a uniform powder. The sample dosage for each test was 5~10 mg, the heating range was from room temperature to 600 °C, and the heating rate was controlled at 10 °C/min. The thermogravimetric (TG) curve and derivative thermogravimetric (DTG) curve of the sample were recorded, and the temperature corresponding to 5% weight loss was taken as the initial thermal decomposition temperature. Meanwhile, the mass-retention rate at different temperature ranges was measured. Three parallel samples were set for each group of experiments, and the average value after removing outliers was taken as the final test result [39].

#### 4.4.8. Micromorphology Characterization (SEM Test)

A field emission scanning electron microscope (FE-SEM) was used to observe the three-dimensional network structure, pore size distribution, and micromorphology characteristics of the gel. The samples were divided into 4 groups: fresh xerogel prepared with the optimal formula, gel after high-temperature aging at 150 °C for 16 h, gel after swelling equilibrium in simulated-formation water, and xerogel of gel-lost circulation particle composite system [40].

All samples were first freeze-vacuum dried for 48 h to a constant weight. The dried samples were fixed on the sample stage with conductive adhesive and subjected to ion sputtering gold-coating treatment with a gold spraying time of 120 s. The accelerating voltage for the test was set at 5 kV, and the micromorphology of the samples was observed at magnifications of 500×, 2000×, and 5000×, respectively. The influence law of the crosslinking density, aging effect, swelling behavior, and lost circulation particles on the microstructure of the gel was analyzed.

#### 4.4.9. Performance Test of Gel-Rigid Lost-Circulation Particle Composite System

Aiming at the engineering pain points of multi-scale fractures and severe lost circulation of large fractures in fractured formations, a gel-rigid lost-circulation particle composite sealing system was constructed based on the optimal basic formula of DF-PG, to clarify the regulation law of particle compounding on the gelation performance, mechanical properties, and sealing performance of the gel [41].

Composite Lost Circulation Particle Formula: Multi-scale rigid lost-circulation materials commonly used in the field were selected, with the compounding ratio of 30% 400-mesh ultrafine calcium carbonate + 20% 800-mesh silica fume + 30% 60-mesh quartz sand + 20% 3 mm polypropylene fiber, taking into account bridging, filling, toughening, and adaptability.

Experimental Design: The optimal formula of DF-PG was used as the blank control group, and the above composite lost-circulation particles with mass fractions of 2%, 4%, 6%, 8%, and 10% were added to the gel base fluid respectively. After stirring evenly, the composite gel system was prepared according to the optimal process, with 3 parallel samples set for each group.

Test Items: Referring to the previous methods, the basic properties of the composite system, including the gelation time, gelation strength, compressive strength, and bonding strength, were tested respectively. The tests focused on the sealing breakthrough pressure, sealing efficiency, and 72 h anti-erosion performance of the system on artificial cores with fractures of different widths from 0.5 mm to 5.0 mm. Meanwhile, the compatibility between the composite system and the field commonly used water-based drilling fluid was tested according to the method in Section 2.4.6, to clarify the optimal compounding ratio and synergistic sealing mechanism.

## Figures and Tables

**Table 1:** Comparison between this system and existing gel systems.

Authors	Temperature Resistance	Compressive Strength	Core Advantages for Fractured Formations
This system	Long-term temperature resistance up to approximately 120 °C	1.2 MPa at room temperature; retains 1.0 MPa after aging at 120 °C	① Compatible with multi-scale composite fractures ② Elastic structure resists long-term erosion mediated by drilling fluid ③ Good compatibility with drilling fluids
Jia et al. [27]	Applicable temperature range: 75–105 °C	The gel with 5% nano-silica only reaches 21 kPa (pure gel: 8.7 kPa), presenting a low strength magnitude	Nano-filler slightly improves the pressure-bearing capacity of the gel; fast gelation rate; chemically degradable
Guo et al. [28]	Sand bed plugging performance stable at 150–180 °C	Plugging breakthrough pressure of 5.3 MPa for 20–40 mesh sand beds	Shear-thinning behavior facilitates injection; strong internal adhesion inside the gel network
Chen et al. [13]	Long-term temperature resistance up to 140 °C	For 3–5 mm fractures, the fluid loss of 5 mm fractures reaches 300 mL under 6 MPa	Stable at 140 °C; excellent pressure-bearing capacity for wide fractures
Du et al. [29]	Narrow gelation window (gelation at 90 °C, gel breaking at 110 °C)	① Breakthrough pressure of 6.8 MPa for 0.5 mm fractures ② Formation damage rate of approximately 10% after gel breaking	Autonomous gelation/gel breaking triggered by temperature; low formation damage

**Table 2:** Fitting parameters of swelling kinetics and water diffusion coefficients of DF-PG gel.

Swelling Medium	Temperature/°C	Diffusion Exponent *n*	Rate Constantk/h^−n^	Water Diffusion Coefficient D/(cm^2^⋅s^−1^)	Goodness of Fit
Deionized water	25	0.52	0.216	3.72 × 10^−7^	0.997
Simulated formation water	25	0.48	0.183	2.95 × 10^−7^	0.996
Simulated formation water	120	0.56	0.274	5.18 × 10^−7^	0.998

**Table 3:** Performance comparison between DF-PG and conventional guar gum LCM.

Property	DF-PG	Guar Gum LCM
Equilibrium swelling ratio (%)	268	185
Compressive strength (MPa)	1.2	0.73
Strength retention after 150 °C aging (%)	82.4	51.7
Plugging efficiency (%)	96.3	84.5
Breakthrough pressure (MPa)	8.7	5.2

**Table 4:** Ion composition of simulated-formation water.

Ion Type	Concentration (mg/L)
Na^+^	7200
Ca^2+^	1200
Mg^2+^	400
Cl^−^	10,800
SO_4_^2^	400

## Data Availability

The data that support the findings of this study are available from the corresponding authors upon reasonable request.

## References

[1] Sun J., Bai Y., Cheng R., Lyu K., Liu F., Feng J., Lei S., Zhange J., Hao H. (2021). Research progress and prospect of plugging technologies for fractured formation with severe lost circulation. Pet. Explor. Dev..

[2] Xu C., Xie J., Kang Y., Liu L., Guo K., Xue D., You Z. (2024). Fracture prepropping and temporary plugging for formation damage control in deep naturally fractured tight reservoirs. SPE J..

[3] Krueger R.F. (1986). An overview of formation damage and well productivity in oilfield operations. J. Pet. Technol..

[4] Xu C., Liu L., Yang Y., Kang Y., You Z. (2024). An innovative fracture plugging evaluation method for drill-in fluid loss control and formation damage prevention in deep fractured tight reservoirs. Fuel.

[5] Hess P.H., Clark C., Haskin C., Hull T. (1971). Chemical method for formation plugging. J. Pet. Technol..

[6] Xu C., Kang Y., Chen F., You Z. (2016). Fracture plugging optimization for drill-in fluid loss control and formation damage prevention in fractured tight reservoir. J. Nat. Gas Sci. Eng..

[7] Lei S., Sun J., Bai Y., Lyu K., Zhange S., Xu C., Cheng R., Liu F. (2022). Formation mechanisms of fracture plugging zone and optimization of plugging particles. Pet. Explor. Dev..

[8] Lu C., Luo Y., Li J., Chen C., Xiao Y., Liu W., Lu H., Guo J. (2020). Numerical analysis of complex fracture propagation under temporary plugging conditions in a naturally fractured reservoir. SPE Prod. Oper..

[9] Zhang R., Hou B., Tan P., Muhadasi Y., Fu W., Dong X., Chen M. (2020). Hydraulic fracture propagation behavior and diversion characteristic in shale formation by temporary plugging fracturing. J. Pet. Sci. Eng..

[10] Wang T., Chen M., Wu J., Lu J., Luo C., Chang Z. (2021). Making complex fractures by re-fracturing with different plugging types in large stress difference reservoirs. J. Pet. Sci. Eng..

[11] Li M., Zhou F., Sun Z., Dong E., Zhuang X., Yuan L., Wang B. (2022). Experimental study on plugging performance and diverted fracture geometry during different temporary plugging and diverting fracturing in Jimusar shale. J. Pet. Sci. Eng..

[12] Pan Y., Cui X., Wang H., Lou X., Yang S., Oluwabusuyi F.F. (2023). Research progress of intelligent polymer plugging materials. Molecules.

[13] Chen J., Qiu H., Djouonkep L.D.W., Lv J., Xie B. (2024). Preparation, evaluation and field application of thermally induced crosslinked polymer gel leakage plugging agent. J. Polym. Environ..

[14] Zhu J., Wang T., Zhang S., Bai Y., Qin G., Yang J. (2025). Study on Gel–Resin Composite for Losting Circulation Control to Improve Plugging Effect in Fracture Formation. Gels.

[15] Liu J., Kou X., Liang X., Gao X., Ye Z., Jin Y. (2024). Research Progress on Leak Prevention and Plugging Technology in Deepwater Drilling. J. Phys. Conf. Ser..

[16] Li C., Yang S., Wen Z., Pan Y., Zeng F., Zhao C., Abouleilah H.M.A. (2024). The research of new type gel plugging agent for deep well. Energy Sources Part A Recovery Util. Environ. Eff..

[17] Fan X., Zhao P., Zhang Q., Zhang T., Zhu K., Zhou C. (2018). A polymer plugging gel for the fractured strata and its application. Materials.

[18] Ying X., Yuan X., Yadong Z., Ziyi F. (2021). Study of gel plug for temporary blocking and well-killing technology in low-pressure, leakage-prone gas well. SPE Prod. Oper..

[19] Xu P., Yu J., Xie L. (2024). Synthesis and Evaluation of Plugging Gel Resistant to 140 °C for Lost Circulation Control: Effective Reduction in Leakage Rate in Drilling Process. Polymers.

[20] Wang Q., Cai J., Wang J., Zhou C., Wen X., Zhang J., Mao H. (2024). Development and application of the anti-high-temperature delayed crosslinking polymer as a gel plugging additive for drilling fluid. Gels.

[21] Zhang S., Wei F., Liu P., Shao L., Li W. (2020). Plugging mechanisms of polymer gel used for hydraulic fracture water shutoff. e-Polymers.

[22] Zhu D.-Y., Fang X.-Y., Sun R.-X., Xu Z.-H., Liu Y., Liu J.-Y. (2021). Development of degradable pre-formed particle gel (DPPG) as temporary plugging agent for petroleum drilling and production. Pet. Sci..

[23] Li J., Tan Z., Jing Q., Mei W., Shen W., Feng L., Dong T., Hao Z. (2025). Research and Performance Evaluation of Low-Damage Plugging and Anti-Collapse Water-Based Drilling Fluid Gel System Suitable for Coalbed Methane Drilling. Gels.

[24] Fang J., Zhang X., Li L., Zhang J., Shi X., Hu G. (2022). Research progress of high-temperature resistant functional gel materials and their application in oil and gas drilling. Gels.

[25] Jia H., Niu C.-C., Yang X.-Y. (2020). Improved understanding nanocomposite gel working mechanisms: From laboratory investigation to wellbore plugging application. J. Pet. Sci. Eng..

[26] Bai Y., Wu L., Luo P., Li D. (2022). Synthesis and evaluation of delayed anti-high temperature gel plugging agent. Front. Energy Res..

[27] Jia H., Yang X., Li S., Yu P., Zhang J. (2020). Nanocomposite gel of high-strength and degradability for temporary plugging in ultralow-pressure fracture reservoirs. Colloids Surf. A Physicochem. Eng. Asp..

[28] Guo C., Jiang G., Guan J., Huang S., Guo Y., He Y., Yang L., Dong T. (2024). Preparation and performance evaluation of a thixotropic polymer gel for loss circulation control. Fuel.

[29] Du G., Peng Y., Pei Y., Zhao L., Wen Z., Hu Z. (2017). Thermo-responsive temporary plugging agent based on multiple phase transition supramolecular gel. Energy Fuels.

[30] Kang Z., Liu Y.-T., Jia H., Xia B., Ou H.-M., Pu Q.-B., Hussein I.A. (2024). Progress and prospects of in situ polymer gels for sealing operation in wellbore and near-well zone. Energy Fuels.

[31] Sarris E.N., Gravanis E. (2022). A hydrodynamic model for analysing the closure stresses inthe Wwellbore strengthening problem. Int. J. Numer. Anal. Methods Geomech..

[32] Sarris E.N., Gravanis E. (2025). Investigating the Role of Plastic and Poroclastoplastic Effectsin Wellbore Strengthening Using a Fully Coupled Hydro-Mechanical Model. Appl. Sci..

[33] Xu C., Kang Y., Chen F., You Z. (2017). Analytical model of plugging zone strength for drill-in fluid loss control and formation damage prevention in fractured tight reservoir. J. Pet. Sci. Eng..

[34] Wang Q., Zhou C., Zhang H., Zhang X., Wen X., Bai J., Mao H. (2024). Preparation of low-molecular-weight polyacrylamide as the delayed crosslinking plugging agent for drilling fluid. Gels.

[35] Niu C., Fan S., Chen X., He Z., Dai L., Wen Z., Li M. (2024). Preparation and performance evaluation of a supramolecular polymer gel-based temporary plugging agent for heavy oil reservoir. Gels.

[36] Cai J., Wu X., Gu S. (2009). Research on Environmentally Safe Temporary Plugging Drilling Fluid in Water Well Drilling. Proceedings of the SPE Asia Pacific Health, Safety, Security, Environment and Social Responsibility Symposium.

[37] Han J., Sun J., Lv K., Yang J., Li Y. (2022). Polymer gels used in oil–gas drilling and production engineering. Gels.

[38] Du J., Wang Q., Liu P., Xiong G., Chen P., Chen X., Liu J. (2023). Nanocomposite gels for water shut-off and temporary plugging in the petroleum industry: A review. Pet. Sci. Technol..

[39] Qu Y., Zhou X., Ren H., Yuan Y., Zhang W., Yang G., Zhang X. (2023). Study on self-healing gel plugging agent based on non-covalent bonding interaction for drilling fluid. J. Appl. Polym. Sci..

[40] Liu Z., Xu J., Peng W., Yu X., Chen J. (2023). The development and deployment of degradable temporary plugging material for ultra-deepwater wells. Processes.

[41] Bai Y.-R., Dai L.-Y., Sun J.-S., Jiang G.-C., Lv K.-H., Cheng R.-C., Shang X.-S. (2022). Plugging performance and mechanism of an oil-absorbing gel for lost circulation control while drilling in fractured formations. Pet. Sci..

